# BRD4: an effective target for organ fibrosis

**DOI:** 10.1186/s40364-024-00641-6

**Published:** 2024-08-30

**Authors:** Qun Wei, Cailing Gan, Meng Sun, Yuting Xie, Hongyao Liu, Taixiong Xue, Conghui Deng, Chunheng Mo, Tinghong Ye

**Affiliations:** 1 Laboratory of Gastrointestinal Cancer and Liver Disease, Department of Gastroenterology and Hepatology, State Key Laboratory of Biotherapy, West China Hospital, Sichuan University, Chengdu, 610041 China; 2 Key Laboratory of Birth Defects and Related Diseases of Women and Children of MOE, State Key Laboratory of Biotherapy, West China Second University Hospital, Sichuan University, Chengdu, 610041 China; 3 Ningxia Medical University, Yin Chuan, 640100 China

**Keywords:** BET family, BRD4, Organ fibrosis, Signaling pathways, BRD4 inhibitor

## Abstract

Fibrosis is an excessive wound-healing response induced by repeated or chronic external stimuli to tissues, significantly impacting quality of life and primarily contributing to organ failure. Organ fibrosis is reported to cause 45% of all-cause mortality worldwide. Despite extensive efforts to develop new antifibrotic drugs, drug discovery has not kept pace with the clinical demand. Currently, only pirfenidone and nintedanib are approved by the FDA to treat pulmonary fibrotic illness, whereas there are currently no available antifibrotic drugs for hepatic, cardiac or renal fibrosis. The development of fibrosis is closely related to epigenetic alterations. The field of epigenetics primarily studies biological processes, including chromatin modifications, epigenetic readers, DNA transcription and RNA translation. The bromodomain and extra-terminal structural domain (BET) family, a class of epigenetic readers, specifically recognizes acetylated histone lysine residues and promotes the formation of transcriptional complexes. Bromodomain-containing protein 4 (BRD4) is one of the most well-researched proteins in the BET family. BRD4 is implicated in the expression of genes related to inflammation and pro-fibrosis during fibrosis. Inhibition of BRD4 has shown promising anti-fibrotic effects in preclinical studies; however, no BRD4 inhibitor has been approved for clinical use. This review introduces the structure and function of BET proteins, the research progress on BRD4 in organ fibrosis, and the inhibitors of BRD4 utilized in fibrosis. We emphasize the feasibility of targeting BRD4 as an anti-fibrotic strategy and discuss the therapeutic potential and challenges associated with BRD4 inhibitors in treating fibrotic diseases.

## Introduction

Fibrosis is an excessive wound-healing process induced by repeated or chronic tissue damage due to persistent external stimuli [1–4]. It can occur in a variety of solid organs or tissues and accounts for 45% of all-cause mortality worldwide [5, 6]. Organ fibrosis, which affects the heart, liver, lungs, and kidneys, is a common complication of chronic diseases such as diabetes mellitus, hypertension, heart disease, and viral or non-viral hepatitis. These conditions can lead to further complications, such as liver cirrhosis, interstitial lung disease, diabetic nephropathy, and heart failure [7]. Currently, pirfenidone and nintedanib are the only drugs approved by the FDA to treat pulmonary fibrosis [8, 9]. However, these drugs have no efficacy in reversing the pathological process of pulmonary fibrosis and have side effects [10–12]. Moreover, there are no clinically approved therapeutic agents for treating cardiac, hepatic, or renal fibrosis. Therefore, it is imperative to find efficient targets for organ fibrosis and to create pharmaceuticals that are therapeutically antifibrotic.

**Fig. 1:**
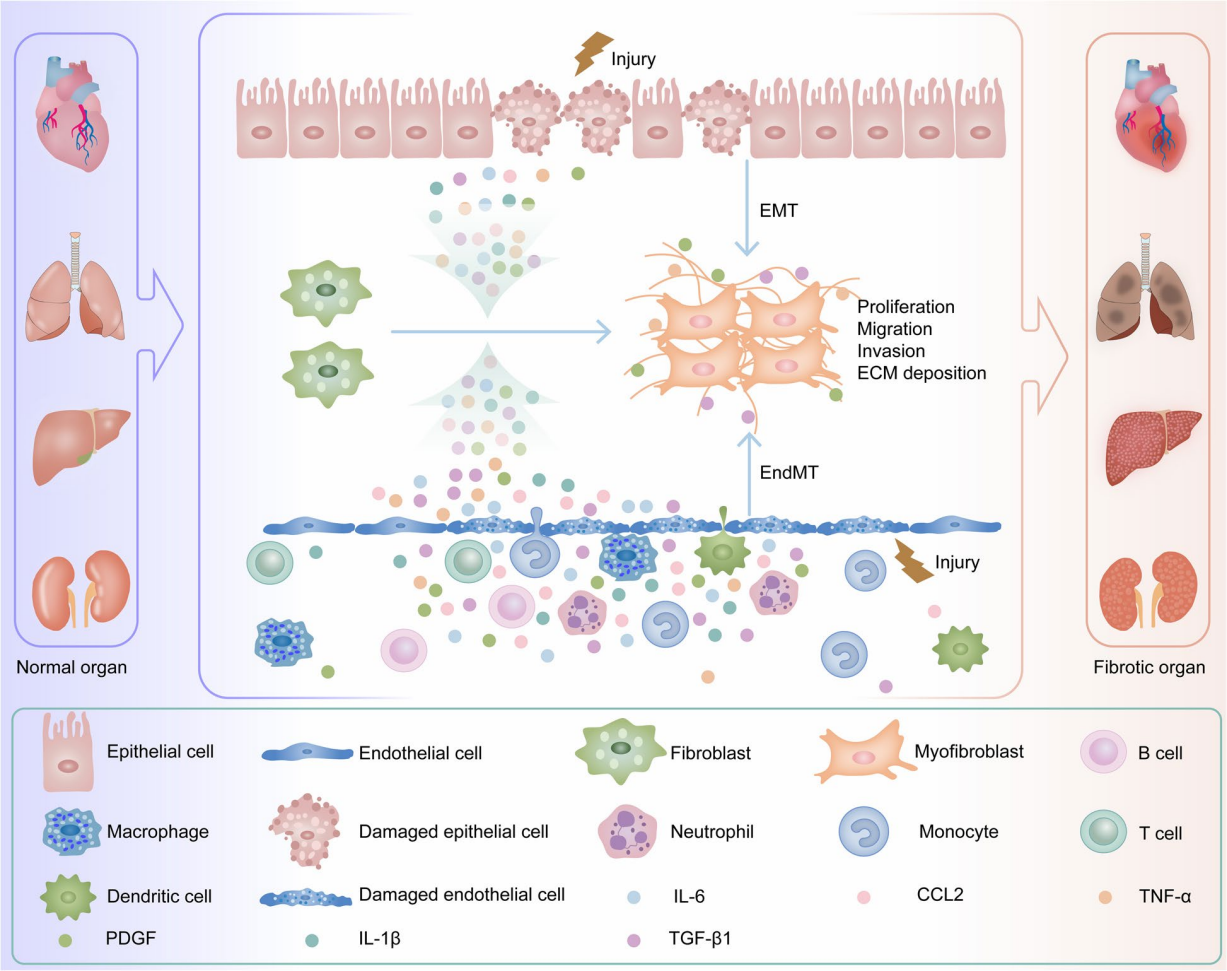
Schematic diagram of the mechanism of organ fibrosis. When tissues and organs such as the heart, liver, lung and kidney are subjected to sustained external injury, damaged parenchymal cells initiate injury-related molecular patterns, secrete inflammatory mediators, recruit inflammatory cells, and promote inflammation in damaged tissues. Quiescent fibroblasts in these organs are activated to become myofibroblasts in response to the body's repair response to damaged tissues, and epithelial-mesenchymal transition (EMT) and endothelial-mesenchymal transition (EndMT) further increase the number of myofibroblasts in damaged organs. Myofibroblasts continuously secrete extracellular matrix (ECM), such as α-smooth muscle actin (α-SMA), collagen I (COL I), and fibronectin (FN), which leads to the development of organ fibrosis [7, 67–70]

Given the enormous threat to human health posed by organ fibrosis, our focus is on the pathological mechanisms underlying its development. When body organs are exposed to external stimuli, damaged cells first undergo necrosis [13]. Damaged cells results in the secretion of inflammatory mediators and the recruitment of inflammatory cells, thus triggering inflammation [14, 15]. Inflammatory cells secrete pro-inflammatory and pro-fibrotic chemokines or cytokines (e.g., transforming growth factor-β1 (TGF-β1), tumor necrosis factor-α (TNF-α), platelet-derived growth factor (PDGF), interleukin-6 (IL-6), interleukin-1β (IL-1β), and C–C motif chemokine ligand 2 (CCL2)) [16]. These factors induce the expression or silencing of fibrosis-related genes in cells surrounding damaged tissues (e.g., endothelial cells, epithelial cells, fibroblasts, hepatic stellate cells (HSCs), pericytes, etc.) [17], which causes precursor cells to transform into myofibroblasts [18]. Activated and proliferating myofibroblasts rapidly synthesize and secrete the extracellular matrix (ECM) [19–23]. After the completion of tissue repair, when the external stimulus is eradicated, the pro-fibrotic mechanism and anti-fibrotic mechanism in the tissue reach a balance [24]. The ECM is remodeled or degraded, and activated myofibroblasts are inactivated through apoptosis, senescence, dedifferentiation, and reprogramming [25]. Parenchymal tissues eventually achieve structural and functional repair, thus completing the normal wound healing process [3]. However, human fibrotic diseases often result from a variety of stimuli. Long-term chronic recurrent tissue injury leads to continuous activation of myofibroblasts and persistent ECM deposition [26]. This leads to gradual escape from the normal biological control of fibrotic repair mechanisms. Excessive ECM disrupts normal tissue structure and physiological function and ultimately leads to organ failure and related complications [27–30] [Fig. 1].

The process of organ fibrosis involves external stimuli that induce the activation of fibroblasts and effector cells. This activation leads to changes in intracellular epigenetics and the expression of fibrotic genes, thereby activating fibrotic signaling pathways and altering the fibrotic phenotypes of cells, tissues, and organs [31, 32]. Epigenetic alterations and the transcription of fibrotic genes are critical in this process [33, 34]. Gene transcription is controlled by interactions between DNA, chromatin modifications, readers, and RNA polymerase II (RNA Pol II) [35–39]. Among chromatin modifications, histone acetylation can regulate chromatin function, thereby disrupting the highly helical structure between DNA and histones. This can modulate the accessibility of genes to the transcriptional machinery and promote the transcription of active genes [40–44]. Histone acetylation is tightly controlled by histone acetyltransferases (HAT) and histone deacetylases (HDAC). HATs include three families, GNAT, MYST, and CBP/P300, and can be considered transcriptional coactivators [45–50]. Histone acetylation marks can be specifically recognized by the epigenetic readout domains of BET families [51–53].

The BET family is a class of epigenetic readers, which are widely studied bromodomain (BRD) proteins whose members are mainly bromodomain-containing protein 2 (BRD2), bromodomain-containing protein 3 (BRD3), BRD4 and bromodomain testis-specific protein (BRDT) [54]. BET family proteins can specifically recognize and bind to acetylated lysine (KAc) residues on histones and nonhistone proteins [55]. This alters chromatin accessibility to transcription factors (TFs), inducing a shift to an active transcriptional state. Furthermore, coactivator proteins involved in transcription initiation and elongation are recruited to histone acetylation marker proteins to promote gene transcription [56–59]. BRD4 is one of the BET family's most thoroughly researched members. It can form transcription complexes in genomic regions with transcription elements such as TFs and transcription cofactor complex mediators [60, 61]. In recent years, BRD4 and BRD4 inhibitors have been extensively studied in the contexts of cancer, inflammation, viral infections, organ fibrosis, etc. [61–66]. The development of BRD4 inhibitors has contributed to the understanding of the role of BRD4 in physiological and pathological processes. Owing to the role of BRD4 in bridging the gap between epigenetic alterations and gene expression, this review focuses on recent progress in understanding the role of BRD4 in organ fibrosis. We introduce the structure and biological functions of BRD4 and summarize BRD4 inhibitors that have been used in organ fibrosis. We further discuss the potential of BRD4 as an anti-fibrotic target, highlighting the therapeutic prospects of BRD4 inhibitors in treating organ fibrosis.

## Structure and function of BET proteins

The four members of the BET family are BRDT, BRD2, BRD3 and BRD4. While BRDT is expressed only in the testis of the male germline, BRD2, BRD3, and BRD4 are universally expressed in all tissues. Two tandem bromodomains (BD1 and BD2) and an extra-terminal (ET) structural domain are present in all BET protein family members. Among them, the bromo-structural domain mainly consists of approximately 110 amino acids. It has four major α-helices (αZ, αA, αB, and αC) in its structure and is connected by two loops (ZA and BC), which link αZ, αA, and αB, αC, respectively [54, 71–73]. These loops form a central hydrophobic pocket that binds specifically to acetylated lysine residues of histones or other proteins [71, 74–76]. The acetylated lysine residues are recognized primarily by asparagine residues in the central hydrophobic pocket [77, 78]. BD1 and BD2 of BRD4 exhibit different binding activities for different acetylated lysine residues [79]. BD1 mainly binds to histone acetylated lysine residues, whereas BD2 mainly binds to other nonhistone acetylated lysine residues [80], such as acetylated lysine residues of RelA and cycle T1 (Cyc T1) [81, 82]. The ET structural domain consists of approximately 80 amino acids [72]. BET proteins mainly recruit TFs or other specific proteins through the ET structural domain to coregulate the transcription of target genes [83, 84].

**Fig. 2:**
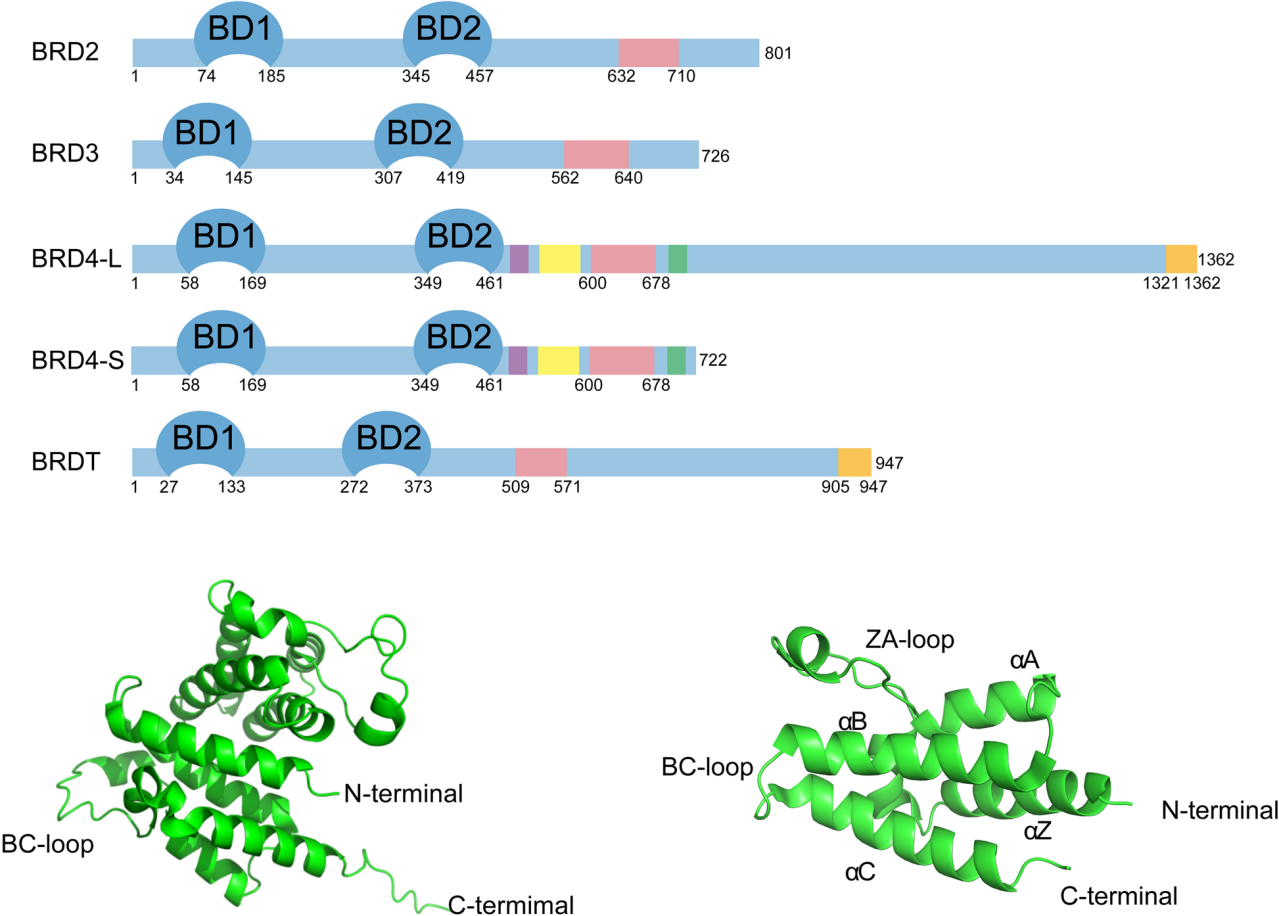
Structures of four members of the human bromodomain and extra-terminal structural domain (BET) family of proteins. All BET proteins have two tandem bromodomains (BD1, BD2) and an extra-terminal structural domain (ET), and the ET structural domains are indicated in pink. There are additional C-terminal domains (CTD), also known as positive transcription elongation factor b (P-TEFb) interaction domain (PID), in the long variant of BRD4 and in the BRDT proteins; CTD structural domains are indicated in orange. The short variant of BRD4 does not have CTD structural domains or histone acetyltransferases (HAT) kinase activity. Additionally, other structural domains were identified in the BRD4 protein, including the N-segment phosphorylation site (NPS), indicated in purple, with an amino acid range of 472–500; the basic residue-enriched structural domain, also known as the basic interaction structural domain (BID), indicated in yellow, with an amino acid range of 524–579; and the C-terminal phosphorylation site (CPS), indicated in green, with an amino acid range of 697–720. Among them, the bromodomain (BD1, BD2) mainly binds to acetylated lysine residues on histones and nonhistone proteins; the ET structural domain mainly mediates interactions with proteins such as transcription factors (TFs); the CTD is mainly responsible for the recruitment of and interaction with P-TEFb; and the NPS is negatively charged, which promotes BD2 binding to acetylated lysine residues, BID is positively charged and can form intramolecular contacts with NPS and inhibit BD2 binding to acetylated lysine residues

In addition, other structural domains have been identified in the BRD4 protein, such as the serine-rich N-terminal phosphorylation site (NPS), which is essential for the binding of BD2 to acetylated lysine residues. The base-interacting structural domain (BID) carries a positive charge and can form intramolecular contacts with the negatively charged phosphorylated NPS, thereby inhibiting the binding of BD2 to acetylated lysine residues [84–86]. BRD4 also contains a serine-rich C-terminal phosphorylation site (CPS) [87, 88]. Furthermore, an additional C-terminal domain (CTD), also referred to as the positive transcription elongation factor b (P-TEFb) interaction domain (PID), is present in the long forms of BRD4 and BRDT and interacts with P-TEFb [89, 90]. P-TEFb is a complex consisting mainly of cell cycle-dependent kinase 9 (CDK9) and Cyc T1 [84, 91]. P-TEFb mostly resides in an inactive form bound to the HEXIM1 protein and the 7SK small nuclear ribonucleoprotein (SnRNP) when it is not bound to BRD4 [92–97]. P-TEFb binding to the CTD of BRD4 inhibits the binding of P-TEFb to HEXIM1 and converts P-TEFb to its active form [98–101]. Ser2 of the RNA Pol II C-terminal motif (CTM) can be phosphorylated by active P-TEFb [101–103], facilitating transcription elongation involving RNA pol II [89, 104–107]. BRD4 also exhibits atypical histone acetyltransferase activity (BRD4-HAT), which acetylates lysine residue 122 on histone H3 (H3K122) [108, 109]. Upon binding to the promoter of the target gene, BRD4 also acetylates lysine residues on neighboring histones, mediating nucleosome dissociation and promoting target gene accessibility for the transcriptional complex [108]. The short variant of BRD4 lacks both intrinsic HAT activity and CTD structural domains [Fig. 2]. In addition, BRD4 was identified as an atypical kinase capable of directly phosphorylating serine 2 (Ser2) of the CTM of RNA pol II which is involved in gene transcription [110].

**Fig. 3:**
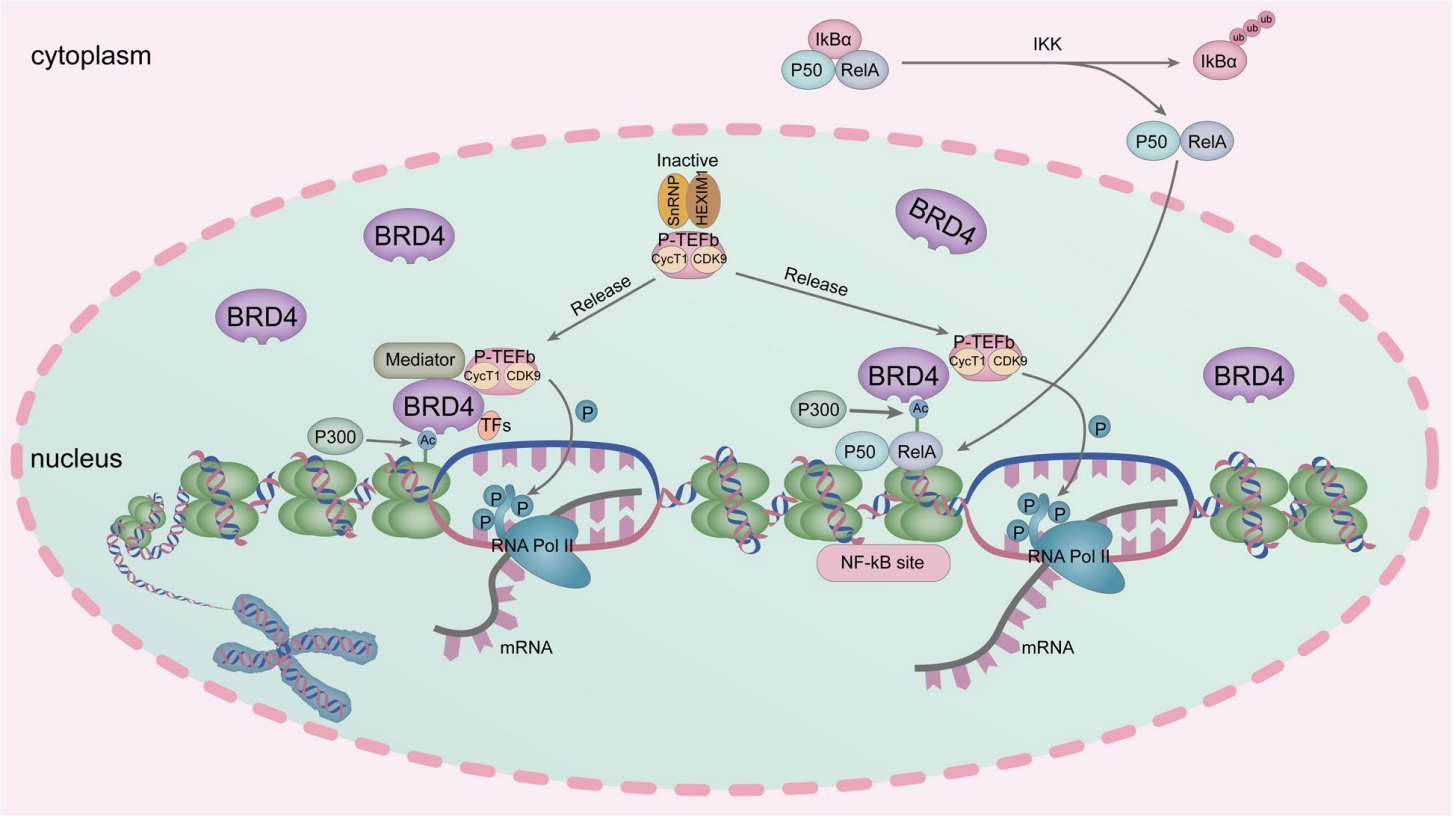
Schematic representation of the function of BRD4. In the presence of histone acetyltransferase (CBP/P300), lysine residues on histones are acetylated, and BRD4 binds to acetylated lysine residues and recruits transcription mediators (Mediator), TFs and P-TEFb. Then Ser2 of the RNA polymerase II (RNA Pol II) C-terminal motif (CTM) is phosphorylated via the cycle-dependent kinase 9 (CDK9) in P-TEFb, facilitating the transcription elongation of RNA Pol II. NF-kB consists of P50 and RelA dissociates from IkBα and translocates to the nucleus. Binds to the promoter of the target gene at the NF-kB site. In the presence of P300/CBP, the lysine at position 310 of RelA is acetylated. BRD4 binds to acetylated RelA and recruits P-TEFb. P-TEFb phosphorylates the CTM of RNA Pol II and promotes the transcription of NF-kB-related inflammatory genes

BRD4 plays a crucial role in the NF-kB-mediated inflammatory gene expression system. NF-kB is a heterodimer consisting of P50 and RelA. Normally, NF-kB is present in the cytoplasm and is bound to the inhibitor IkBα [111–114]. External stimuli lead to the phosphorylation and subsequent degradation of IkBα by triggering the activation of IkB kinase [115–117]. This process releases NF-κB from the inactive complex, allowing it to translocate to the nucleus [118]. Once inside the nucleus, NF-kB binds to NF-kB sites at the promoter of the target gene [119]. The histone acetyltransferase P300/CBP acetylates lysine 310 on RelA, a modification crucial for gene activation [120–123]. Acetylated lysine residues on RelA are recognized and bound by BRD4, which then recruits P-TEFb [117, 124]. P-TEFb phosphorylates the CTM of RNA Pol II via the kinase activity of CDK9 [124, 125]. This promotes the transcription of inflammatory target genes associated with NF-kB [112]. Additionally, BRD4 promotes lysine 310 acetylation of RelA through its atypical acetyltransferase activity, which mediates inflammatory gene transcription [84][Fig. 3].

## BRD4 in organ fibrosis

### BRD4 in cardiac fibrosis

Cardiac fibrosis is a prevalent pathophysiological process in a variety of cardiac diseases, including heart failure, myocardial infarction (MI), hypertensive heart disease, aortic stenosis, hypertrophic cardiomyopathy, dilated cardiomyopathy (DCM), and diabetic cardiomyopathy [126]. Cardiac fibroblasts (CFs) are the primary effector cells of the heart that respond to various external stimuli leading to pathological damage [127–129]. In the process of myocardial injury caused by external stimuli such as myocardial hypoxia, pressure overload, volume overload [126], apoptosis and necrosis of cardiomyocytes, infiltration of inflammatory cells, and activation of CFs gradually occur in damaged foci of the heart, initiating the process of cardiac repair [130]. Sustained external stimulation leads to overactivation of CFs and excessive ECM deposition, inducing cardiac fibrosis. Multiple signaling pathways, including the TGF-β signaling pathway, the renin–angiotensin–aldosterone system [131], endothelin, etc., are involved in CFs activation as well as in the process of cardiac pathological remodeling. Cardiac fibrosis leads to cardiac tissue stiffness [132], ventricular diastolic-systolic dysfunction [133], and impaired electrical conduction, which can trigger cardiac dysfunction and heart failure [126, 134, 135].

Matthew S. Stratton et al. Demonstrated via RNA sequencing and mass spectrometry that BRD4 in CFs is significantly enriched in a subpopulation of promoters, active enhancers, or superenhancers associated with fibrosis in response to stimulation by external factors such as TGF-β and transverse aortic constriction (TAC). This in turn regulates CFs activation in vitro and in vivo. BRD4 function is partially regulated by p38 mitogen-activated protein kinase (MAPK) [136]. Fortunately, Matthew S. Stratton et al. reported that microRNA-9 is able to target the 3'UTR of the BRD4 transcript. MicroRNA-9 was able to suppress BRD4 expression in normal cardiomyocytes. Downregulation of microRNA-9 due to external stimuli leads to increased BRD4 expression and enrichment at distal superenhancers [137]. These findings suggest that BRD4 might mediate the progression of cardiac fibrosis. Given that BRD4 has an epigenetic reader function and transcriptional complex recruitment capacity. We found that BRD4 promotes the expression of cardiac pathology biomarkers by interacting with P-TEFb, facilitating transcription elongation and suspending RNA Pol II release. Importantly, BET inhibitors have been shown to significantly reduce RNA Pol II phosphorylation levels and attenuate its transcriptional elongation [138, 139]. Thus, we believe that BRD4 inhibition could play a therapeutic role in cardiac fibrosis. Isoprenaline (ISO) stimulation promotes PARylation of the BRD4 CTD structural domain by poly (ADP-ribose) polymerase-1 (PARP1). This modification promotes BRD4 binding to the transcriptional start sites of hypertrophic genes, such as atrial natriuretic peptide (ANP) and brain natriuretic peptide (BNP), leading to increased RNA Pol II phosphorylation and hypertrophic gene transcriptional activation [140]. Dingyan Zhou et al. reported that ISO was able to induce the acetylation of H3K122 and the phosphorylation of RNA Pol II. Bellidifolin (BEL), the active xanthone component of gentian, inhibits the protein levels of BRD4 and NADPH oxidase 4 (NOX4), and reduces ISO-induced acetylation of H3K122 and phosphorylation of RNA Pol II. This led to a reduction in the expression of the cardiac hypertrophy marker proteins ANP and BNP in the mouse heart, which in turn ameliorated myocardial hypertrophy as well as cardiac fibrosis [141].

Numerous researchers have investigated the therapeutic benefits and mechanisms of action of BRD4 inhibitors in heart disease because BRD4 functions in controlling gene expression in heart disease. As described below, BRD4 inhibition has been shown to play a therapeutic role in cardiac fibrosis caused by various types of cardiac injury. First, Shuai Song et al. demonstrated that BRD4 is upregulated in TAC mouse cardiac endothelial cells and in TGF-β-treated mouse cardiac endothelial cells. BRD4 promotes the migration of human umbilical vein endothelial cells (HUVECs) and mouse aortic endothelial cells (MAECs) and induces endothelial-mesenchymal transition (EndMT) in endothelial cells by regulating the expression of EndMT-associated TFs, such as *Snail*, *Slug* and *Twist*, as well as the TGF-β/Smad signaling pathway. The BRD4 inhibitors JQ1 and BRD4 knockdown significantly reversed the EndMT process in in vivo and ex vivo endothelial cells; blocked the synthesis of ECM proteins, such as α-smooth muscle actin(α-SMA), collagen I (COL I), and collagen III (COL III); and slowed cardiac fibrosis while preserving cardiac function [142]. Additionally, Zhangxu He et al. synthesized C-34, an inhibitor that targets BRD4 at the molecular and cellular levels. They discovered that by preventing the activation of the TGF-β/Smad signaling pathway, C-34 significantly reduces the proliferation and migration of neonatal rat cardiac fibroblasts (NRCFs) caused by angiotensin II (Ang II). This inhibition reduces the synthesis of ECM proteins, such as α-SMA, COL I and COL III, both in vivo and ex vivo. Consequently, C-34 effectively alleviates CFs activation and cardiac fibrosis in vivo, improving cardiac function [143]. These studies highlight the therapeutic potential of BRD4 inhibitors in treating cardiac fibrosis by targeting the molecular mechanisms underlying fibroblast activation and ECM synthesis. Deletion of the LMNA gene encoding laminin A/C in mouse cardiomyocytes is a common cause of DCM. It leads to cardiac dysfunction, fibrosis, ventricular arrhythmias, and other cardiac disorders. Gaelle Auguste demonstrated that treatment with the BRD4 inhibitor JQ1 partially reverses the transcript levels of molecular markers of cardiac hypertrophy and cardiac dysfunction in LMNA-deficient cardiomyocytes. It also reduces or normalizes the transcript levels of genes involved in fibrosis in the hearts of LMNA mice, ameliorates cardiac fibrosis, improves cardiac function, and prolongs the survival of cardiomyocyte-specific deletion LMNA mice [144]. These findings underscore the therapeutic potential of BRD4 inhibitors, such as JQ1, in treating various forms of cardiac fibrosis and dysfunction by targeting key molecular pathways involved in fibrosis and inflammation.

Inflammation is a pivotal factor in the progression of cardiac fibrosis. As indicated earlier, the interaction between BRD4 and NF-kB is important for regulating the expression of genes associated with inflammation. Research has demonstrated that upon stimulation with TNF-α, activated NF-κB rapidly enters the nucleus and recruits BRD4 to enhancers and promoters in the genome. The binding of BRD4 to NF-kB establishes a superenhancer regions and promotes the transcription of proinflammatory genes in a BRD4-dependent manner [145]. These findings suggest that BRD4 inhibition could provide indirect therapeutic benefits in cardiac fibrosis by ameliorating inflammation. Andrew Antolic et al. found in a mouse model of DCM that BRD4 was positively correlated with inflammatory genes induced by NF-kB in CFs. BRD4 mediates the expression of NF-kB-related inflammatory gene networks and fibrotic signaling networks through direct interaction with RelA. The BRD4 inhibitor JQ1 significantly suppressed CFs activation, adverse cardiac remodeling, and the extent of cardiac fibrosis [146]. Furthermore, Yiping Sun et al. confirmed that under hypoxic conditions, BRD4 binds to acetylated RelA in cardiomyocytes and is significantly recruited to the transcriptional promoters of NPPA and NPPB [147]. Interestingly, the inhibition of BET proteins has been found to mitigate cardiac damage in rats experiencing acute MI by modulating the TLR4/TRAF6/NF-kB pathway. This intervention reduces the enrichment of inflammatory cells in the infarcted area, improves cardiac remodeling, and reduces the infarct size [148]. Richard J. Mills et al. reported that the BET inhibitor INCB054329 blocked lipopolysaccharide (LPS)-induced proinflammatory cytokine production and exerted a preventive effect against inflammation-induced cardiac dysfunction in vitro and in vivo [149]. The BRD4 inhibitor JQ1 can upregulate silent information regulator 1 (SIRT1) expression in vitro and in vivo*.* This effect ameliorates LPS-induced cardiac inflammation and oxidative stress by inhibiting SIRT1-dependent activation of NOD-like receptor protein 3 (NLRP3) inflammatory vesicles [150]. JQ1 also enhances myocardial autophagy via the liver kinase β1 (LKB1)-AMPK-ULK1 signaling pathway; inhibits the release of inflammatory factors such as IL-6, IL-1β, TNF-α and interferon-γ (IFN-γ); and prolongs the survival time of allogeneic heart grafts [151]. Notably, Jinxia Han et al. showed that fucoxanthin (FX) inhibited Ang II-induced CFs transdifferentiation. This may be related to the fact that FX down-regulated the expression of BRD4 in CFs, reversed the inhibitory effect of Ang II on the downstream antioxidant signaling pathway of BRD4, nuclear factor erythroid 2-related factor 2 (Nrf2)/heme oxygenase-1(HO-1), and inhibited the increase in reactive oxygen species (ROS) and oxidative stress in CFs [152]. Another study revealed that during cardiac hypertrophy, ROS promoted BRD4 expression in mouse cardiomyocytes. BRD4 inhibition significantly reduced the prohypertrophic effects of Ang II on cardiomyocytes and significantly decreased the gene expression of fibrotic markers such as *Tgfb1*, *Col1a1*, *Col1a3* and *Ctgf*. This also reversed the increase in P-NF-kB expression and the decrease in superoxide dismutase (SOD) activity caused by Ang II and aortic banding (AB) surgery while increasing the mRNA levels of Nrf-2 and HO-1. These findings suggest that BRD4 inhibition exerts cardioprotective effects through NF-κB signaling and the Nrf-2/HO-1 pathway [153]. These studies suggest that BRD4 inhibition is also capable of exerting an anti-cardiac fibrosis effect through oxidative stress pathways.

**Fig. 4:**
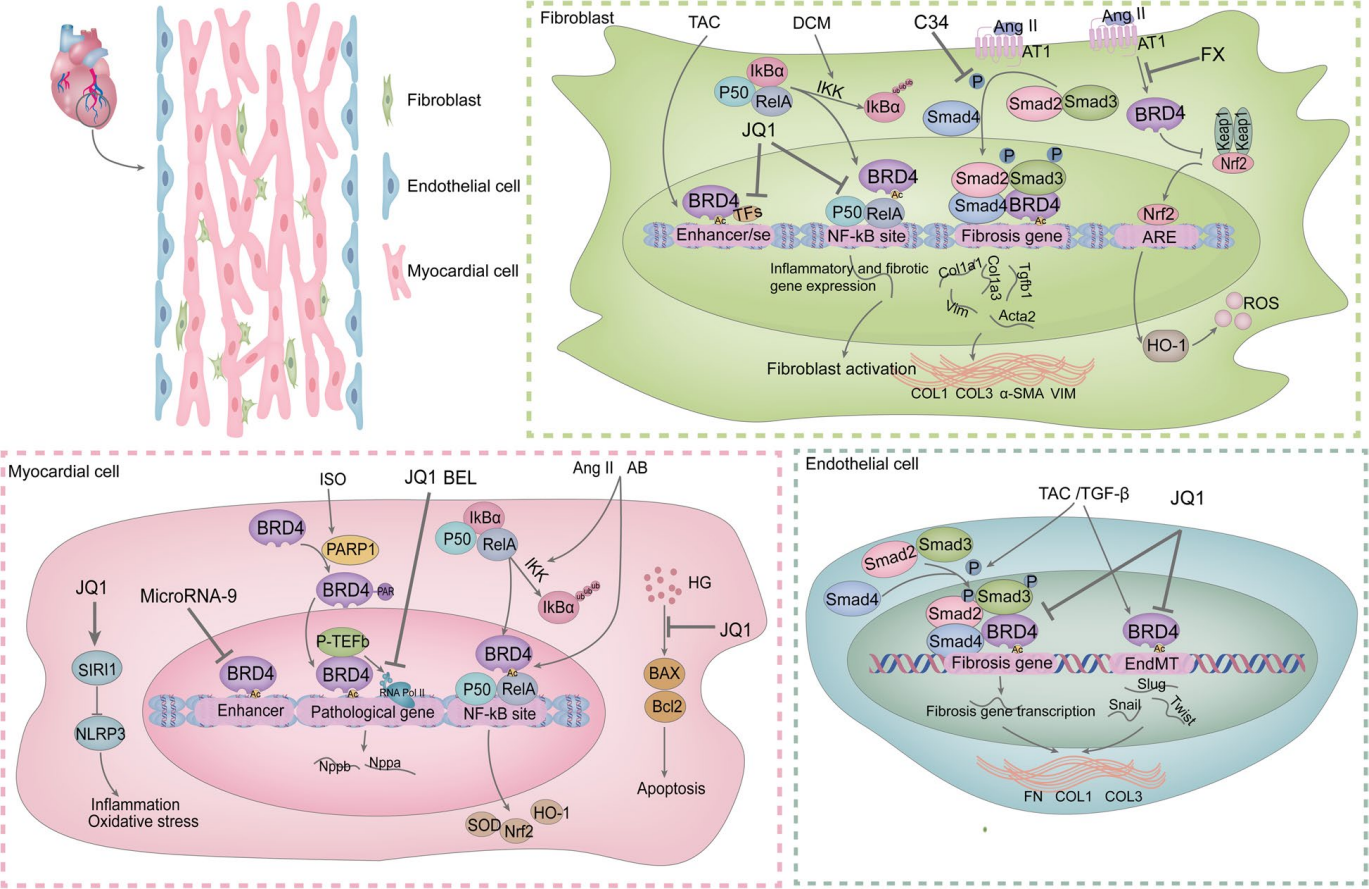
Mechanisms of cardiac fibrosis involved in BRD4 and anti-cardiac fibrosis effects and mechanisms of BRD4 inhibitors. BRD4 is involved in fibrotic phenotypic changes in CFs, cardiomyocytes, and endothelial cells caused by external stimuli. It mainly affects the expression of fibrosis-related genes. BRD4 inhibitors (JQ1, C34, FX, and BEL) exert their anti-cardiac fibrosis effects mainly by inhibiting BRD4-mediated gene transcription, the TGF-β/Smad signaling pathway and oxidative stress

Myocardial fibrosis and apoptosis are significant features of diabetic cardiomyopathy. Hyperglycemia leads to the activation, proliferation, migration and synthesis of multiple ECM proteins in CFs and promotes the apoptosis of cardiomyocytes (H9C2) [154]. Relevant studies have confirmed that hyperglycemic stimulation elevates BRD4 expression levels in both H9C2 cells and CFs. The BRD4 inhibitor JQ1 can act on the TGF-β1/SMAD family member 3 (SMAD3) and protein kinase B (AKT) signaling pathways, attenuating the expression of fibrogenic genes such as *Tgf-β*, *Ctgf*, *Col1α1*, *Smad3*, and other fibrogenic genes in high-glucose (HG)-induced CFs. It also reduces the BAX/Bcl2 ratio in H9C2 cells, thereby exerting anti-fibrosis and anti-apoptotic effects [154, 155]. In summary, BRD4 inhibition can exert anti-cardiac fibrosis effects by modulating multiple pathways related to fibrosis, inflammation and oxidative stress in cardiac effector cells, such as myocardial fibroblasts, cardiomyocytes and cardiac endothelial cells [Fig. 4].

### BRD4 in pulmonary fibrosis

Idiopathic pulmonary fibrosis (IPF) is a progressive, lethal, age-related lung disease [156–161]. The development of IPF involves multiple pathological processes. External stimuli causes damage to alveolar epithelial cells and subsequent lung inflammation [162–164]. Multiple cytokines in damaged lesions induce epithelial-mesenchymal transition (EMT) and fibroblast activation, which increases the proportion of myofibroblasts in the lungs and promotes myofibroblast synthesis of ECM and tissue repair [165–167]. However, when external stimuli persist, continuous damage to alveolar epithelial cells, ongoing lung inflammation, and persistent myofibroblast activation drive excessive and uncontrolled synthesis of the ECM [168, 169]. This leads to excessive deposition of ECM in the lung interstitial space, resulting in destruction of the alveolar structure and severe impairment of gas exchange, which can ultimately cause respiratory failure and death [170–172]. Currently, only pirfenidone and nintedanib are approved by the FDA for the treatment of pulmonary fibrosis [173–175]. However, these drugs can slow disease progression but cannot stop or reverse it.

The critical role of the transdifferentiation and dedifferentiation of myofibroblasts in the progression of pulmonary fibrosis has been recognized by numerous researchers. Ksenija Bernau et al. used ZL0591, a highly selective variant inhibitor capable of binding specifically to BRD4 BD1, to assess the role of BRD4 in myofibroblast transdifferentiation. The i*n vivo* results revealed that ZL0591 reversed the increase in lung radiodensity after bleomycin (BLM) treatment and reduced the levels of α-SMA, *Vim*, *Tnc*, and *Col1α1* in fibrotic lung tissues. These findings confirm that BRD4 BD1 inhibitors inhibit myofibroblast transdifferentiation and attenuate BLM-induced pulmonary fibrosis [176]. Consistent with these findings, KenicHi SuZuKi et al. found in vitro that JQ1 significantly inhibited the expression levels of α-SMA and fibronectin (FN) genes and proteins in myofibroblasts extracted from the lungs of patients with end-stage IPF. This inhibition induced the dedifferentiation of lung myofibroblasts. The manner in which JQ1 exerts its effects may involve the suppression of differentially expressed genes within signaling pathways associated with the ECM and fibrosis while promoting the expression of genes associated with glutathione metabolism [177]. Additionally, Seidai Sato et al. evaluated the therapeutic effect of ARV-825, a novel BRD4 degrader, on pulmonary fibrosis. They confirmed that ARV-825 reduced the expression of BRD4 and senescence markers and induced apoptosis in senescent cells. Moreover, ARV-825 inhibited the expression of fibrosis-associated proteins in TGF-β-stimulated nonsenescent cells, thereby attenuating BLM-induced pulmonary fibrosis [178]. These studies confirm that BRD4 is involved in the transdifferentiation of lung myofibroblasts and the progression of pulmonary fibrosis, suggesting its potential as a therapeutic target for pulmonary fibrosis.

What are the specific pathways by which BRD4 participates in the process of pulmonary fibrosis? Shunya Kaneshita et al. found that BRD4 was highly expressed in BLM-induced lung fibrotic lesions. Subsequent investigations demonstrated that within primary fibroblasts obtained from fibrotic mouse lungs, BRD4 exhibited notable enrichment in the promoter regions of the thrombospondin 1 (*Thbs1)*, integrin β3 (*Itgb3)*, and *Acta2* genes. This enrichment promoted autocrine/paracrine TGF-β1 and pro-fibrotic actions associated with TGF-β1. In addition, the administration of CG223, a quinolone BRD4 inhibitor designed and synthesized by Shunya Kaneshita, significantly attenuated BLM-induced pulmonary fibrosis. It also decreased the mRNA levels of *Thbs1*, *Itgb3*, and *Acta2* induced by TGF-β1 in primary lung fibroblasts (LFs), with the strength of the effect being dose dependent. Additionally, it decreases the expression of COL I, COL III, and α-SMA in myofibroblasts [179]. Similarly, TGF-β1 promotes the acetylation of H4K5 and the aggregation of BRD4 at the promoter regions of IL-6, plasminogen activator inhibitor-1 (PAL-1) and α-SMA. These findings suggest that TGF-β1 and BRD4 play complementary roles in the progression of pulmonary fibrosis. Xiaoyan Tang et al. found that the BET inhibitor JQ1 attenuated the TGF-β1 and PDGF-BB mediated migration and proliferation of LFs and reduced the expression of COL I and IL-6. The same results were obtained using BRD4 knockout [180]. Therefore, BRD4 inhibition could block the TGF-β1 signaling pathway to a certain extent, thus exerting an anti-pulmonary fibrosis effect. More excitingly, Bing Tian et al. discovered that polyinosinic:polycytidylic acid (poly I:C) could induce EMT in human small airway epithelial cell lines (hSAECs) [181]. BRD4 plays a role in stabilizing the transcription elongation complex during this process. Inhibitors of BRD4 significantly disrupt BRD4-HAT and RelA complex formation, preventing myofibroblast proliferation and attenuating fibrosis [182]. In addition, JQ1 treatment disrupted the enrichment of RelA, CDK9, and RNA Pol II at the promoters of EMT-associated TFs, such as *SNAI1*, *ZEB1*, *IL-6*, and *VIM.* This suppressed the expression of mesenchymal genes, such as *SNAI1*, *FN1*, and *VIM*, induced by repetitive TGF-β1 stimulation. This inhibited TGF-β-induced epithelial fibrosis, expansion of mesenchymal supporting cells, thickening of alveolar septa and increased hydroxyproline content in the lungs of mice [183]. Other in vivo findings have shown that the BET inhibitor JQ1 also inhibits TGF-β1-dependent gene expression and BRD4 binding at the Gli1 promoter region in cancer-associated fibroblasts (CAFs), reducing fibroproliferation and cancer cell proliferation [184]. Taken together, BRD4 contributes to pulmonary fibrosis disease progression by participating in multiple signaling pathways involved in TGF-β/Smad signaling.

The ROS-generating enzyme NOX4 and the antioxidant enzyme SOD2 are central to the regulation of intracellular ROS. Moreover, redox dysregulation is a key factor in the induction of LFs activation. Previous studies have shown that the gene expression of *NOX4* is significantly increased, whereas the expression of *SOD2* is significantly inhibited in human fibrotic LFs. Further research revealed that BRD4 binds to the NOX4 promoter and is involved in the TGF-β-induced gene expression of NOX4. This mechanism explains the ability of the BRD4 inhibitors JQ1 and OTX015 to inhibit the binding of BRD4 to the NOX4 promoter and reduce the enrichment of H4K16ac and P300 at the NOX4 gene promoter. This resulted in reduced protein and gene expression of NOX4 in primary IPF LFs and in TGF-β1-induced normal human embryonic LFs (IMR90). Additionally, these inhibitors inhibit the TGF-β-induced increase in *NOX4* and *ACTA2* and the reduction in *SOD2* while increasing Nrf2 activity, thereby reducing the level of ROS in cells and fibroblast activation and promoting the regression of pulmonary fibrosis in mice [185, 186]. Collectively, this evidence suggests that BRD4 is involved in promoting NOX4 expression and mediating oxidative stress-induced pulmonary fibrosis. Like its effects on cardiac fibrosis, JQ1 was found to reduce the number of inflammatory cells in bronchoalveolar lavage fluid (BALF) in a dose-dependent manner, thereby reversing the inflammation and fibrosis progression in the lungs caused by BLM treatment [187]. These findings suggest that BRD4 inhibition similarly ameliorates the progression of fibrosis in the lungs by attenuating inflammation. Interestingly, Bing Tian et al. found that repetitive mucosal cat dander extract (rCDE) stimulation triggered BRD4-HAT and phosphorylated RNA Pol II kinase activity by activating NF-kB/RelA, leading to the acetylation of Lys122 on histone H3 [188]. Airway fibrosis, EMT and myofibroblast expansion induced by rCDE are dependent on BRD4 function. Treatment with Zl0454, a small molecule inhibitor of BRD4, reduced mucosal H3K122 acetylation accumulation and the expression of snail family transcriptional repressor 1 (SNAI1), vimentin (VIM), FN, COL I, and α-SMA [188]. However, the exact mechanisms by which BRD4 inhibition ameliorates pulmonary fibrosis by suppressing inflammation remain to be further explored.

**Fig. 5:**
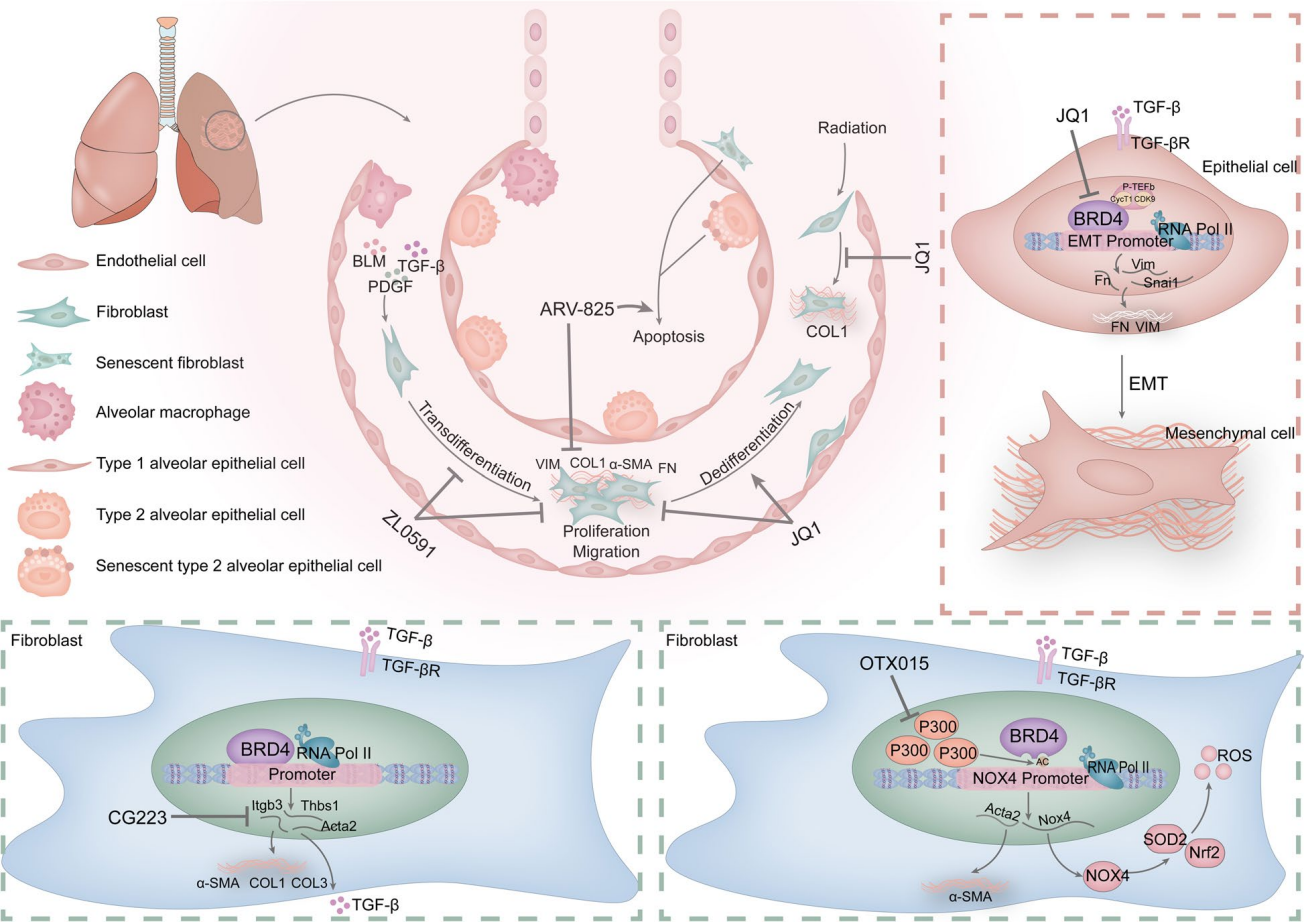
Mechanisms of pulmonary fibrosis involved in BRD4 and anti-pulmonary fibrosis effects and the mechanisms of BRD4 inhibitors. BRD4 is involved in fibrotic phenotypic changes in lung fibroblasts (LFs) and epithelial cells caused by external stimuli. It mainly affects the expression of fibrosis-related genes. BRD4 inhibitors (JQ1, ZL0591, CG233, OTX015, and ARV-825) exert their anti-pulmonary fibrosis effects mainly through the inhibition of BRD4-mediated gene transcription, the activation of LFs, the EMT process and oxidative stress

BET protein inhibition is also a therapeutic for radiation-induced lung fibrosis (RILF). Chunshan Liu et al. found that iBET-BD2 (GSK046) is an inhibitor that selectively targets the second bromodomain of BET proteins. It was able to attenuate radiation-induced fibroblast fibrin marker expression [189]. Similarly, Jian Wang et al. demonstrated that JQ1 administration significantly attenuated inflammatory infiltration, fibrosis and collagen deposition in the lungs caused by irradiation. Further assays revealed that JQ1 administration reduced the radiotherapy-induced expression of BRD4, c-MYC, COLI, TGF-β, P65, and p-Smad2/3. The transdifferentiation of human LFs to myofibroblasts resulting from radiation treatment was similarly inhibited by JQ1 [190]. Furthermore, in addition to its significant involvement in inflammation, oxidative stress, and fibrosis in pulmonary fibrosis, BRD4 has also been associated with various other lung-related diseases. Notably, BRD4 expression levels are elevated in the blood and sputum of patients with chronic obstructive pulmonary disease (COPD). BRD4 was positively correlated with the expression of IL-6 and interleukin-8 (IL-8) in bronchial epithelial cells (BEAS-2B cells) following exposure to cigarette smoke and viral infection [191]. MiR-218 attenuated cigarette smoke extract-mediated secretion of the inflammatory factors TNF-α, IL-6, and IL-8 in bronchial epithelial cells by targeting and inhibiting the expression of BRD4 [192]. Zhen Xiao et al. demonstrated that JQ1 may delay the progression of COPD by increasing the expression of agrin protein [193]. In addition, JQ1 blocked the nuclear translocation and acetylation of P65 and inhibited the binding of P65 to DNA in the lung tissues of mice with COPD. JQ1 administration improved inflammation, oxidative stress, and lung function in the lung tissues of mice in a dose-dependent manner, exerting a protective effect against COPD [194, 195]. During TLR3-NFkB/RelA-mediated airway remodeling, BRD4 inhibitors block the pericyte-to-myofibroblast transition, protecting vascular homeostasis and reducing vascular leakage [196]. BRD4 expression is elevated in patients with idiopathic pulmonary arterial hypertension (PAH) and promotes the upregulation of polo-like kinase 1 (PLK1) via forkhead box M1 (FoxM1). RVX208 inhibits proliferation, promotes apoptosis, ameliorates inflammation through the BRD4-FoxM1-PLK1 axis in PAH-associated vessels, and improves vascular remodeling and pulmonary hemodynamics in rats in vivo [197]. In summary, we concluded that BRD4 can serve as a target in pulmonary fibrosis and has the potential to serve as a therapeutic target in lung diseases such as airway remodeling, interstitial lung disease, PAH and COPD [198]. BRD4 inhibition effectively mitigates the progression of pulmonary fibrosis and COPD while also ameliorating PAH and acute lung injury [Fig. 5].

### BRD4 in hepatic fibrosis

Chronic alcoholic liver disease, inflammation, hepatitis viral infection, cholestasis and many other diseases that cause liver injury can induce hepatic fibrosis [69, 199, 200]. Hepatic fibrosis eventually progresses to cirrhosis or hepatocellular carcinoma (HCC), leading to liver failure and death if the onset and progression of hepatic fibrosis are not controlled in a timely manner. HSCs are the main precursor cells of myofibroblasts in the liver and are quiescent under physiological conditions [201, 202]. Liver injury induced by various external stimuli results in hepatocyte necrosis and cytokine release [203]. This allows HSCs to be activated to gain the ability to proliferate, migrate and synthesise ECM for tissue repair. Long-term liver injury leads to permanent activation of myofibroblasts, which is out of the control of the body's repair mechanism. This results in continuous secretion of ECM and excessive deposition of ECM gradually destroys the normal tissue structure and physiological function of the liver. As a result, liver tissue is gradually replaced by fibrous tissue, leading to the progressive formation of abnormal scar tissue and liver remodeling [204].

Several studies have shown that BRD4 is abnormally elevated in hepatic fibrosis tissues caused by various etiologies such as viral hepatitis infections (hepatitis C virus (HBV), hepatitis C virus (HCV)), non-alcoholic steatohepatitis (NASH), autoimmune hepatitis (AIH), primary biliary cholangitis (PBC), cholestasis, and alcohol-associated liver disease (ALD) [205, 206]. BRD4 has been detected predominantly in hepatocytes, activated HSCs [207], macrophages and biliary cells. The expression of BRD4 is positively correlated with the severity of hepatic fibrosis as well as alanine transaminase (ALT) and aspartate transaminase (AST) levels in HBV-induced hepatic fibrosis [206]. These findings suggest that BRD4 may be involved in the development of hepatic fibrosis. Research has demonstrated that BRD4 has the capacity to facilitate the progression of liver fibrosis via various pathways. First, Ning Ding et al. demonstrated that BRD4 binds to the *COL1A1* enhancer site and is significantly enriched at the motifs of pro-fibrotic TFs, such as ETS proto-oncogene 1 (ETS1), serum response factor (SRF), SMAD3, and NF-kB. BRD4 is able to participate in the transcriptional regulation of pro-fibrotic genes through enhancer progenitors. In addition, BRD4 is able to directly target the PDGF pathway as a mitogenic regulator of HSCs proliferation. The presence of JQ1 inhibits the expression of genes involved in cell activation during primary HSCs activation, suggesting that BRD4 has the potential to be an anti-fibrosis target [208]. Second, similar to its role in pulmonary fibrosis, BRD4 synergistically promotes with the TGF-β signaling pathway in hepatic fibrosis. Feifan Xu et al. found that BRD4 is involved in the effects of TGF-β1 on the PDGF receptor and the SMAD3, signal transducer and activator of transcription 3 (STAT3) and AKT pathways. TGF-β1-induced increases in the expression of histone acetyltransferase P300, NF-kB P65, and tissue inhibitor of metalloproteinase 1 (TIMP1) require the participation of BRD4 [209]. At the same time, TGF-β1 significantly upregulated the protein and gene expression levels of BRD4 in HSCs. The mechanism involves TGF-β1-induced activation of the STAT3 signaling pathway, which promotes the expression of the early-immediate gene (Egr1). TGF-β1 further facilitates the binding of Egr1 to the BRD4 promoter, thereby promoting the expression of BRD4, leading to HSCs activation and hepatic fibrosis. Furthermore, Egr1 also has a positive feedback effect on STAT3 activation. Knockdown of BRD4 blocks the induction of HSCs activation and hepatic fibrosis via STAT3 signaling [210]. In terms of other signaling pathways, Xiaobo Cai et al. found that BRD4 is involved in the activation of HSCs bychemokine (C-X-C motif) ligand 6 (CXCL6). CXCL6 was able to enhance the interaction of SMAD3 with BRD4, BRD4 with the C-MYC promoter, and C-MYC with the enhancer of zeste homolog 2 (EZH2) promoter via the SMAD2/BRD4/C-MYC/EZH2 pathway, inducing the activation of quiescent HSCs into myofibroblasts [211]. Additionally, Jinhang Gao et al. demonstrated that BRD4 is involved in TNF-α-induced P300/NF-κB/BRD4 transcriptional activator complex formation and *CCL2* transcription. TNF-α leads to acetylation of histone H3K27 in the enhancer and promoter regions of the *CCL2* gene via P300. Increased *CCL2* expression with the involvement of the epigenetic reader BRD4 leads to hepatic fibrosis and portal hypertension [212]. Miao Cheng et al. further confirmed that BRD4 knockdown in mice with CCl_4_-induced hepatic fibrosis significantly reduced the degree of fibrosis and suppressed the expression of fibrosis-associated genes. This mechanism may be related to the involvement of P300 in the PLK1 promoter in H3K27 acetylation, and BRD4 promotes PLK1 expression by recognizing and binding to acetylated H3K27 [213]. Moreover, BRD4 also binds to acetylated P65 (lysine 310) in the NF-kB signaling pathway in mouse macrophages while exerting a pro-inflammatory effect [214]. Additionally, USP22, a deubiquitylating protease, is able to interact with and reduce the ubiquitylated level of BRD4 to promote the inflammatory response in ALD involving BRD4 [205]. Taken together, these findings suggest that BRD4 can serve as an effective target against hepatic fibrosis.

**Fig. 6:**
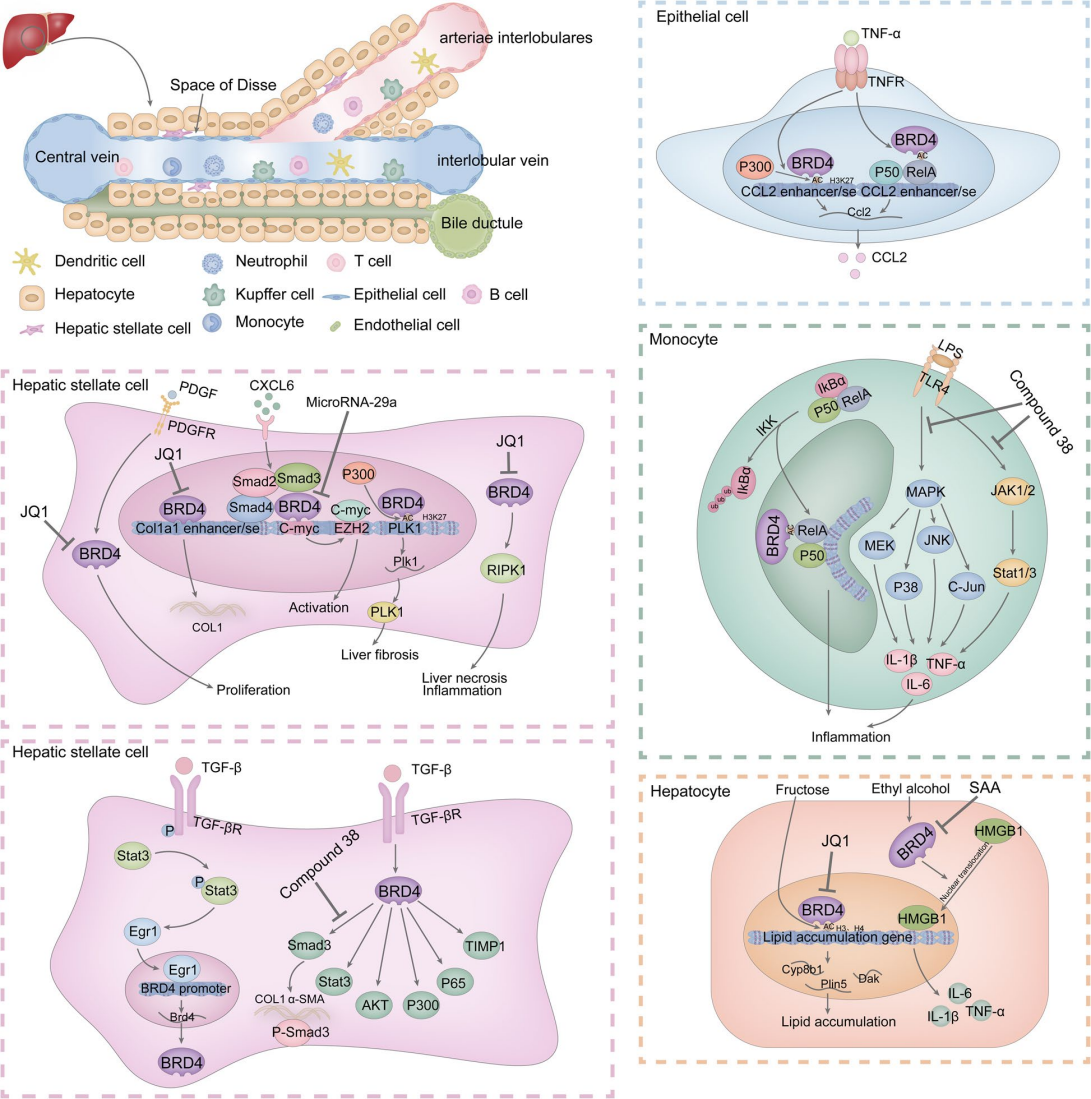
Mechanisms of hepatic fibrosis involved in BRD4 and anti-hepatic fibrosis effects and mechanisms of BRD4 inhibitors. BRD4 is involved in fibrotic phenotypic changes in hepatic stellate cells (HSCs), epithelial cells, macrophages and hepatocytes caused by external stimuli. It mainly affects the expression of fibrosis-related genes. BRD4 inhibitors (JQ1, Compound 38, SAA) exert their anti-hepatic fibrosis effects mainly by inhibiting BRD4-mediated gene transcription, activation of HSCs, and expression of inflammatory factors

JQ1 is a classical small molecule inhibitor of BRD4. JQ1 has been found to reduce BRD4 enrichment at the *COL1A1* enhancer site, decrease the expression of pro-fibrotic genes in HSCs, inhibit their activation, and prevent and reverse CCl_4_-induced fibrosis in the mouse liver [208]. Additionally, JQ1 was able to bind the bromodomain of P300 and inhibit its acetyltransferase activity. In turn, it treats S. japonicum-infected induced hepatic fibrosis in mice by inhibiting RORγt acetylation and decreasing the expression of Th17-specific cytokines [215]. Hepatic injury is a predisposing factor for hepatic fibrosis. JQ1 attenuates hepatic necrosis, inflammation and mitochondrial dysfunction by inhibiting the expression of receptor-interacting protein kinase 1 (RIPK-1) through the BRD4/RIPK1 axis [216]. Abnormalities in lipid metabolism contribute to the development of hepatic fibrosis. Aki Yamada and colleagues reported that excessive fructose intake led to increased acetylation of histones H3 and H4. This facilitated the enrichment of BRD4 in the transcriptional areas of genes associated with lipid accumulation (*Cyp8b1*, *Dak*, and *Plin5*), which increased expression of genes linked to lipid accumulation. JQ1 treatment attenuated the effects of fructose overdose on the above pathological processes [217]. Another study showed that JQ1 upregulated PD-L1 expression in HCC cells by increasing Rab8A expression, thus promoting the immunotherapeutic effect of an anti-PD-L1 antibody on HCC cells [218]. In addition to JQ1, other BRD4 inhibitors have been used to treat hepatic fibrosis. Sarah A. Middleton et al. evaluated the effects of the small molecule inhibitor of the BET protein GSK1210151A (I-BET151) on NASH and hepatic fibrosis. They found that I-BET151 treatment was able to maintain glucose homeostasis, reduce the expression of inflammatory genes in the interferon signaling pathway, and inhibit the expression of *Tgf-β*, *Timp1* and other fibrosis-related genes [219]. Rong Fu et al. synthesized a BET inhibitor, Compound 38, based on ABBV-075 (one of the most potent inhibitors of the BET bromodomain). They found that compound 38 attenuated inflammatory cell infiltration in the liver as well as the expression of pro-inflammatory factors such as IL-6, TNF-α, and IL-1β in macrophages via the JAK-STAT and MAPK signaling pathways. It further alleviated the liver injury and inflammation caused by LPS/GalN injection. Meanwhile, compound 38 inhibited the activation of HSCs and exerted antifibrotic effects through the TGF-β/SMAD and Wnt/β-catenin signaling pathways [220]. Yanwen Lan et al. reported that salvianolic acid A (SAA), an active ingredient in Salvia miltiorrhiza, could effectively act on the BRD4/high mobility group box 1 (HMGB1) pathway. It inhibited the translocation of HMGB1 by down-regulating BRD4 expression, reduced the expression of ethanol-induced inflammatory factors such as IL-6, IL-1β, and TNF-α, and attenuated ethanol-induced liver injury [221]. Simplicity acid (SA) is a phenolic acid that binds specifically to BRD4 and alleviates alcohol-induced liver injury by attenuating oxidative stress and pyroptosis [222]. In addition, Ying Hsien Huang et al. demonstrated that microRNA-29a overexpression reduced BRD4 and SNAI1 expression in a BDL-induced hepatic fibrosis model. MicroRNA-29a overexpression was also able to down-regulate the expression of EZH2 and SNAI1 in primary HSCs and increase peroxisome proliferator-activated receptor-γ (PPAR-γ) expression, which inhibited the migration and proliferation of HSCs [223]. The down-regulation of BRD4 by microRNA-29a was further confirmed by Yen-Cheng Lin et al. [224]. HCC is the result of the progression of hepatic fibrosis to the end stage. Cho-Hao Line et al. reported that a bivalent BRD4 inhibitor, AZD5153, was able to inhibit the expression of pro-carcinogenic genes associated with BRD4, inhibit the proliferation of hepatocellular carcinoma cells and promote their apoptosis [225]. In summary, BRD4 can serve as an effective target for treating hepatic fibrosis, and BRD4 inhibition can significantly attenuate the progression of hepatic fibrosis through TGF-β, inflammation and other signaling pathways [Fig. 6].

### BRD4 in renal fibrosis

Chronic kidney disease (CKD) is a chronic fibrotic disease that results from the progression of renal fibrosis to the later stages of the disease. Renal fibrosis is the ultimate pathological process shared by chronic kidney injury or adaptive repair [226]. Oxidative stress [227, 228], inflammation [229], lupus nephritis [230], hypertensive nephropathy (HN) [231], diabetic nephropathy [232], acute kidney injury (AKI) [233], and renal ischemia–reperfusion injury (IRI), among many other renal disorders, can lead to renal injury, which can trigger renal fibrosis. Myofibroblasts are the primary producers of ECM in the kidneys [234]. When the kidneys are subjected to various external stimuli, pericytes [235, 236], mesenchymal stem cell-like cells, macrophages [237, 238], epithelial cells [239], and perivascular fibroblasts in the kidneys transform into myofibroblasts. These myofibroblasts participate in the repair of damaged kidney tissues and produce large amounts of ECM, leading to the onset of renal fibrosis [240, 241].

Several studies have shown that BRD4 is upregulated in a variety of renal diseases, including hyperuricemia nephropathy [242], focal segmental glomerulosclerosis (FSGS), LgA nephropathy [243], renal IRI [244], unilateral ureteral obstruction(UUO) renal injury [245], HN [246], diabetic kidney disease (DKD) [247], and renal cell carcinoma (RCC) [248]. These findings indicate that BRD4 might be involved in the pathogenesis of various renal diseases and could serve as a promising therapeutic target for managing renal fibrosis. In terms of renal fibrosis and kidney injury, progressive glomerulonephritis (GN) is characterized by excessive deposition of collagen IV (COL IV) in the glomeruli and glomerulosclerosis. José Luis Morgado Pascual et al. examined the effect of JQ1 on GN in a murine model of nephrotoxic serum (NTS)-induced anti-glomerular basement membrane nephritis. JQ1 attenuated NTS-induced accumulation of COL IV in glomeruli and glomerulosclerosis and alleviated UUO-induced sex-determining region Y-box 9 (SOX9) nuclear translocation and renal interstitial fibrosis. Mechanistically, JQ1 can block the TGF-β1/Smad signaling pathway, attenuate the binding of BRD4 to the *Col4a3* promoter, and inhibit the activation and nuclear translocation of SOX9 in the kidneys of NTS-treated mice [249]. Similarly, Sandra Rayego Mateos et al. showed that JQ1 inhibited the expression of pro-fibrotic factors (TGF-β, connective tissue growth factor (CTGF), PAI-1) and the progression of renal fibrosis in UUO-treated kidneys. JQ1 reduced ECM-associated components (FN and COL I) in TGF-β-treated mesangial cells and renal fibroblasts and inhibited the nuclear translocation of SOX9 [250]. Additionally, JQ1 inhibited the expression of α-SMA, COL III, and FN at the gene and protein levels in Ang II-induced HN kidneys. It also reversed EMT and attenuated Ang II-induced renal injury and renal fibrosis [246]. Maria Laura Saiz et al. encapsulated JQ1 in liposomes and found that liposome-loaded JQ1 attenuated IRI-induced AKI, inflammation and renal fibrosis and improved pharmacokinetics and toxicity [251]. Inflammation is a significant contributor to the pathogenesis of renal fibrosis, and a variety of renal inflammatory diseases are predisposing factors for renal fibrosis. As mentioned above, BRD4 can directly bind to RelA and induce the release of inflammatory factors associated with the NF-kB pathway. BRD4 can also bind directly to the gene promoters of *CCL2*, *CCL5*, and *IL-6*, mediating the expression of pro-inflammatory factors. Suarez Alvarez, Beatriz et al. demonstrated that JQ1 hindered the interaction between BRD4 and the promoters of RelA and genes associated with inflammation. JQ1 administration counteracted TNF-α-induced increases in the pro-inflammatory factors CCL2, C–C motif chemokine ligand 5 (CCL5) and IL-6 in human renal tubular epithelial cells (HK2 cell line). Moreover, JQ1 reduced the infiltration of inflammatory cells and the expression of inflammatory cytokines, chemokines (C–C motif chemokine ligand 20 (CCL-20), chemokine (C-X-C motif) ligand 16 (CXCL-16), and adhesion molecules (intercellular adhesion molecules (ICAM-1)) in a renal fibrosis model and a renal inflammation model. Additionally, JQ1 suppressed Th17 immune responses in renal inflammation, leading to decreased renal inflammation and injury, improved glomerular lesions, and the restoration of renal function [252]. Zhong Gui Gong et al. found that cadmium (Cd) promotes the acetylation of lysine at position 310 in RelA and the binding of BRD4 to acetylated RelA K310, inducing the activation of the NF-kB inflammatory signaling pathway. JQ1 treatment or BRD4 knockdown significantly inhibited the Cd-induced nuclear translocation of NF-kB in vitro and in vivo*.* This further attenuated the transcription of inflammatory cytokines (IL-1β, IL-6, TNF-α, and monocyte chemotactic protein-1 (MCP-1)) in Cd-treated rat kidneys and NRK-52E cells, thereby attenuating Cd-induced inflammation in rat kidneys [253]. In addition, BRD4 knockdown or JQ1 treatment inhibited the phosphorylation of NF-kB and promoted NLRP3 transcription through the NF-kB/NLRP3/caspase-1 signaling pathway. NLRP3 is able to mediate caspase-1-dependent pyroptosis in RCC cells, thereby inhibiting the proliferation and EMT of RCC cells [248]. In terms of oxidative stress, BRD4 is involved in TGF-β1-induced oxidative stress and fibrogenic gene expression. Baoshang Zhou et al. demonstrated that JQ1 inhibited *Nox4* transcription through the TGF-β/Smad signaling pathway and the extracellular signal regulated kinase 1/2 (ERK1/2) pathway, thereby attenuating NOX4-mediated oxidative stress. Furthermore, JQ1 was able to reduce α-SMA and FN deposition in the renal mesenchyme caused by UUO and prevent the progression of renal fibrosis in rats [245]. BRD4 also promotes forkhead box protein O4 (FOXO4) transcription by inhibiting the phosphorylation of the PI3K/AKT signaling pathway. This promotes FOXO4-induced oxidative stress and contributes to hypoxia-reoxygenation (HR) in vitro and to IRI in vivo mediated apoptosis and endoplasmic reticulum stress (ERS). BRD4 inhibition via JQ1 or BRD4 siRNA protects against kidney injury caused by renal IRI [244]. Liping Sun et al. found that JQ1 treatment significantly inhibited cisplatin-induced apoptosis in renal proximal tubular cells and AKI. This mechanism may be related to the fact that JQ1 attenuates CHK2-mediated DNA damage and reduces cisplatin-induced P53, as well as the phosphorylation of P38, ERK1/2, and c-Jun N-terminal protein kinase (JNK) in the MAPK signaling pathway. Interestingly, JQ1 treatment reversed the induction of the antioxidant proteins Nrf2 and HO-1 by cisplatin and inhibited the expression of inducible nitric oxide synthase (iNOS) in nitrosative stress, thereby attenuating cisplatin-induced oxidative stress in the kidney [254]. Cd promoted the acetylation of H4K16, which is involved in autophagy and lysosomal gene expression. It also promoted BRD4 enrichment at the histone H4K16 locus and inhibited the transcriptional levels of lysosomal genes. This leads to lysosomal dysfunction and autophagy blockage, which further leads to oxidative stress and cytotoxicity. JQ1 or BRD4 knockdown significantly restored lysosomal-mediated autophagy and reduced Cd-induced oxidative stress and cytotoxicity, thereby protecting against Cd-induced AKI [255]. In summary, BRD4 plays an important role in promoting renal fibrosis. BRD4 may be an effective target for treating renal fibrosis. The BRD4 inhibitor JQ1 can exert anti-renal fibrosis effects through three pathways: attenuation of renal injury, inflammation and oxidative stress.

**Fig. 7:**
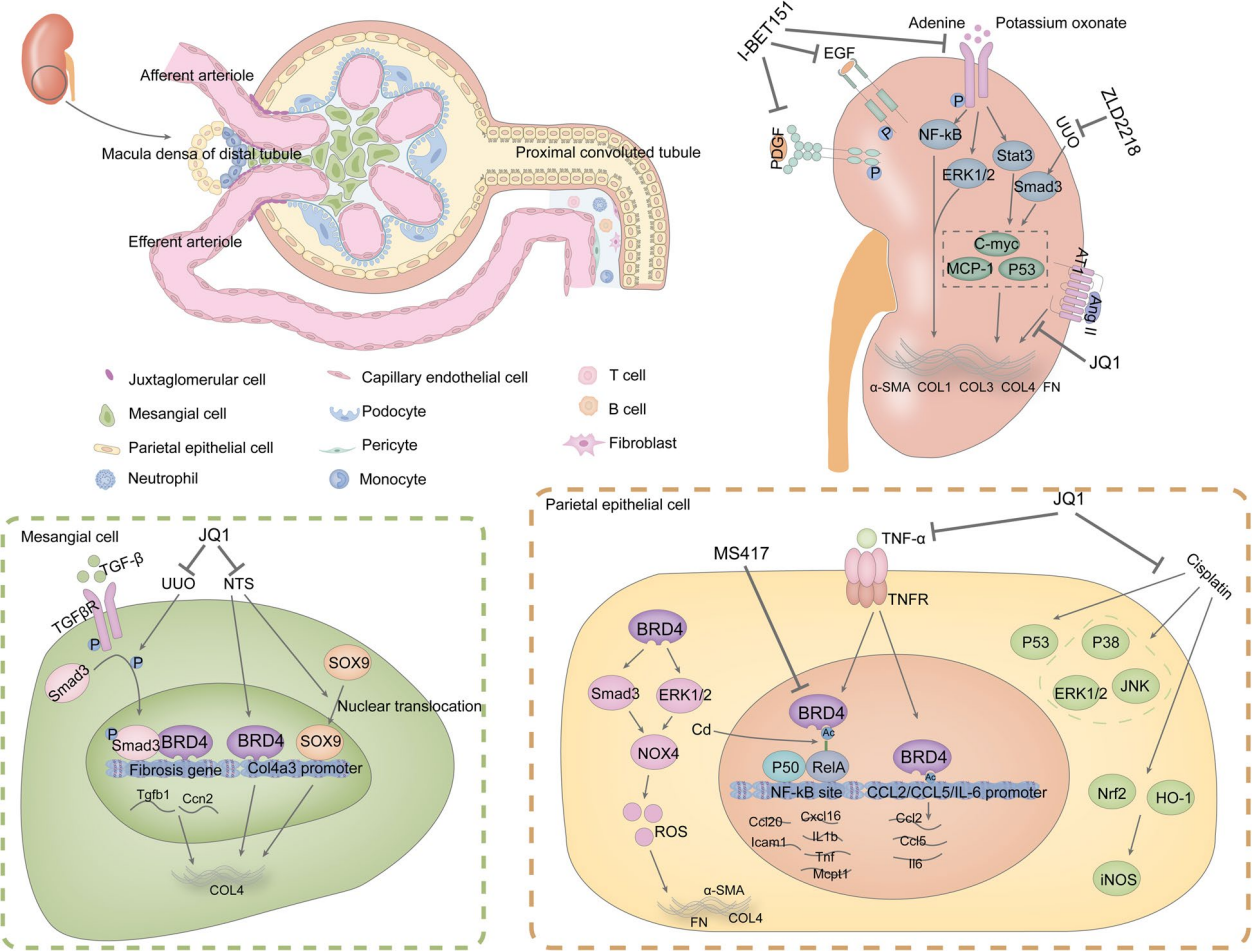
Mechanisms of renal fibrosis involved in BRD4 and anti-renal fibrosis mechanisms of BRD4 inhibitors. BRD4 is involved in the fibrotic phenotypic changes in tethered cells and parietal epithelial cells caused by external stimuli. BRD4 inhibitors (JQ1, I-BET151, ZLD2218, and MS417) mainly inhibit BRD4-mediated gene transcription and oxidative stress to exert anti-renal fibrosis effects

Given the importance of BRD4 in the development of renal fibrosis, researchers have investigated the therapeutic effects of other BRD4 inhibitors for renal disease. Chongxiang Xiong et al. evaluated the therapeutic effects of IBET151, a small molecule inhibitor of the BET protein, on renal fibrosis. They found that while down-regulating the renal levels of BRD4, I-BET151 effectively suppressed the activation of various signaling pathways, including the TGF-β/Smad3, STAT3, ERK1/2, and NF-kB pathways, and inhibited the phosphorylation of epidermal growth factor receptor (EGFR) and platelet-derived growth factor receptor (PDGFR). Additionally, I-BET151 decreased the expression of C-MYC, TNF-α, MCP-1, and P53 and reversed EMT in the kidneys. I-BET151 further reduces the activation of renal mesenchymal fibroblasts and the deposition of α-SMA, COL I and FN, ameliorating renal fibrosis [242, 243]. HIV infection in the kidney induces NF-kB activation, which triggers inflammation. Guangtao Zhang et al. reported that the BRD4 inhibitor MS417 can competitively bind tightly to the bromodomain of BRD4 and block the binding of BRD4 to NF-kB. This inhibited the transcriptional activation of pro-inflammatory cytokines and chemokines related to the NF-kB pathway in the kidney, such as *CCL2*, *CCL3*, *CCL5*, *CCL20*, and *TLR-2.* MS417 also inhibited the expression of pro-apoptotic genes, such as NF-kB-targeted *BCL2*, *FAS*, and *TRAF1*, and attenuated inflammatory cell infiltration and glomerular and tubular injury caused by HIV infection [256]. Meanwhile, MS417 inhibited oxidative stress-induced IL-6 expression and significantly reduced CCL2 expression and the number of centrocytes in the kidneys of IRI mice, thereby attenuating tubular damage caused by IRI [257]. Sibei Tao et al. reported a new BRD4 inhibitor, ZLD2218. In vivo administration of ZLD2218 at 30 mg/kg/day for eight consecutive days significantly inhibited BRD4 levels in fibrotic kidneys. Importantly, ZLD2218 blocked UUO-induced activation of the TGF-β/Smad3 signaling pathway and suppressed α-SMA, COL I, COL IV and FN expression, significantly attenuating renal fibrosis and renal injury [258]. Apabetalone (RVX-208), an oral BET inhibitor used for treating cardiovascular disease, has also shown promise for treating renal disease. Sylwia Wasiak et al. demonstrated that RVX-208 downregulated CKD-related protein markers and molecular pathways in the plasma of renal injury patients. It reduces IL-6, interleukin-17 (IL-17), IL-12 and other plasma protein levels of inflammation-related cytokines and down-regulated the levels of plasma proteins associated with the NF-kB signaling pathway in CKD patients [259]. Min Wang et al. found that BRD4 can activate the NLRP3 inflammasome through the P300/H3K27ac/PLK1 axis, which in turn triggers cellular death and inflammation in the kidney. RVX-208 down-regulates the protein level of BRD4 and attenuates renal injury and fibrosis in DKD [247]. The above studies further confirmed that BRD4 can be a promising target against renal fibrosis and that BRD4 inhibition has a therapeutic effect on renal fibrosis [Fig. 7].

## BRD4 inhibitors in organ fibrosis

To date, there are approximately 1700 inhibitors that target BRD4. Given the role of BRD4 in the development of fibrogenesis, BRD4 inhibitors have significant potential for use in treating organ fibrosis. However, only approximately a dozen BRD4 inhibitors are currently used in preclinical studies of organ fibrosis. Here, we summarize the BRD4 inhibitors that are currently being used in studies of organ fibrosis. The aim of this review was to provide a reference for the in-depth application of BRD4 inhibitors in organ fibrosis.

RVX208 is an orally administered quinazolinone BET inhibitor [260]. In preclinical studies, RVX-208 has been shown to ameliorate PAH and DKD [197, 247]. Early clinical studies focused on exploring the safety of RVX-208 in healthy individuals and patients (NCT00768274, NCT01058018) and its therapeutic efficacy in PAH (NCT03655704). RVX208 has now progressed to clinical phase III trials for reducing adverse cardiovascular events in patients with type 2 diabetes mellitus and coronary artery disease (NCT02586155) [261]. OTX015 (MK-8628), a triazolo benzodiazepine BET inhibitor [262], is currently in clinical trials to explore its appropriate dose range in patients with hematological malignancies (NCT01713582) and advanced solid tumors (NCT02259114) [263–269]. In organ fibrosis, OTX015 was able to inhibit the binding of BRD4 and *NOX4* promoters, reducing ROS levels in fibroblasts and attenuating pulmonary fibrosis in mice [186]. JQ1, the first BET inhibitor, is a thieno-triazolo-1,4-diazepine synthesized by Panagis Filippakopoulos et al. in 2010. JQ1 binds highly specifically to the bromodomain of the BET family, occupying the acetylated lysine-binding pocket of BRD4 and interfering with BRD4 and histone acetylated lysine binding [270]. In organ fibrosis, JQ1 has been extensively used to explore the role of BRD4 in fibrotic disease progression. Current studies have shown that JQ1 is able to exert therapeutic effects on organ fibrosis through various pathways, such as reversing the EndMT and EMT processes, blocking the TGF-β and NF-kB signaling network pathways, reversing the oxidative stress imbalance, inhibiting BRD4 binding to the *NOX4* promoter, inhibiting myofibroblast activation, inhibiting BRD4 enrichment at the promoters of fibroblastic genes, and reducing inflammatory cytokine expression. I-BET151 is an imidazolidinone-based selective inhibitor of BET [271, 272], which has good bioavailability in rats and minipigs [273]. In preclinical studies, I-BET151 has been shown to inhibit the activation of the TGF-β/Smad3, STAT3, ERK1/2, and NF-kB signaling pathways in renal fibrosis and to reverse renal EMT [242, 243]. In hepatic fibrosis, I-BET151 is able to maintain glucose homeostasis and reduce the expression of inflammatory genes in the interferon signaling pathway, thus exerting a therapeutic effect on hepatic and renal fibrosis [219]. MS417, an inhibitor of BRD4, blocks the binding of BRD4 to acetylated RelA, thereby inhibiting the expression of genes associated with the NF-kB pathway [256, 274, 275]. In CKD, MS417 hinders the transcriptional activation of pro-inflammatory cytokines and chemokines linked to the NF-kB pathway, leading to a decrease in the expression of IL-6 and CCL2, thereby attenuating the infiltration of inflammatory cells and glomerular injury [257]. C-34, a phenylquinazoline-based BRD4 inhibitor synthesized by Wen Zhao's group, has been shown to block the activation of the TGF-β1/Smad2/3 signaling pathway, inhibit fibroblast activation, and ameliorate cardiac fibrosis [143]. CG223, a quinolone-based BET inhibitor, inhibits the TGF-β signaling pathway to exert an anti-fibrotic effect on pulmonary fibrosis [179]. ZL0454, a BRD4-selective inhibitor with cyclopentylbenzenesulfonamide scaffolding developed by Allan R. Brasier et al., blocks the NF-kB signaling pathway and attenuates inflammatory responses triggered by external stimuli, improving airway remodeling [188, 276]. ZL0591 was synthesized by Zhiqing Liu et al. and is a selective mutational inhibitor that targets BRD4 BD1. The in vivo half-life of ZL0591 was significantly prolonged relative to that of ZL0454 [277]. ZL0591 has been shown to significantly ameliorate pulmonary fibrosis [176]. ARV-825 is a novel BET degrader. As a heterobifunctional protein hydrolyzes targeting chimeras, it can effectively induce the binding of E3 ubiquitin ligases to the BET family and degrade BET family proteins [278]. ARV-825 has demonstrated good preclinical antifibrotic effects against pulmonary fibrosis [178]. Compound 38 is a derivative optimized on the basis of the structure of ABBV-075 by Zizhou Li et al. [279]. It is capable of attenuating the hepatic inflammatory response through the JAK-STAT and MAPK signaling pathways. In addition, compound 38 also exerts anti-hepatic fibrotic effects through the TGFβ/SMAD and Wnt/β-catenin signaling pathways [220]. ZLD2218 is a BRD4 inhibitor of pyrrolidinones, which was obtained by Sibei Tao et al. on the basis of the structural optimization of ABBV-075, INCB057643, I-BET151 and PLX51107, and it was able to significantly reduce BRD4 levels in fibrotic kidneys and block the TGF-β/Smad3 signaling pathway to exert an anti-kidney fibrotic effect [258]. In summary, these BRD4 inhibitors have shown significant potential in treating various forms of organ fibrosis [Table 1]. Continued studies of these inhibitors could further elucidate the therapeutic potential of BRD4 in fibrotic diseases.

**Table 1:** BRD4 fibrosis inhibitor summary table

Compound name	Compound structure	Applications in diseases	clinical progress
RVX-208		Pulmonary arterial hypertension[197], Chronic kidney disease [259], Diabetic kidney disease [247]	Phase 3 Clinical
OTX015(MK-8628)		Pulmonary fibrosis [186]	Phase 2a clinical
JQ1		Cardiac fibrosis[142], Renal fibrosis [245], Pulmonary fibrosis [183, 190], Liver fibrosis [208]	preclinical
I-BET151(GSK1210151A)		Liver fibrosis[219], Renal fibrosis [243], Hyperuricemic nephropathy [242]	preclinical
MS417		Renal ischemia reperfusion injury (IRI)[257], Diabetic nephropathy (DN) [275]	preclinical
C-34		Cardiac fibrosis [143]	preclinical
CG223		Pulmonary fibrosis [179]	preclinical
ZL0454		Airway remodeling [188]	preclinical
ZL0591		Pulmonary fibrosis [176]	preclinical
ARV-825		Pulmonary fibrosis [178]	preclinical
Compound 38		Liver fibrosis[220]	preclinical
ZLD2218		Renal fibrosis [258]	preclinical

## Conclusions and perspectives

**Fig. 8:**
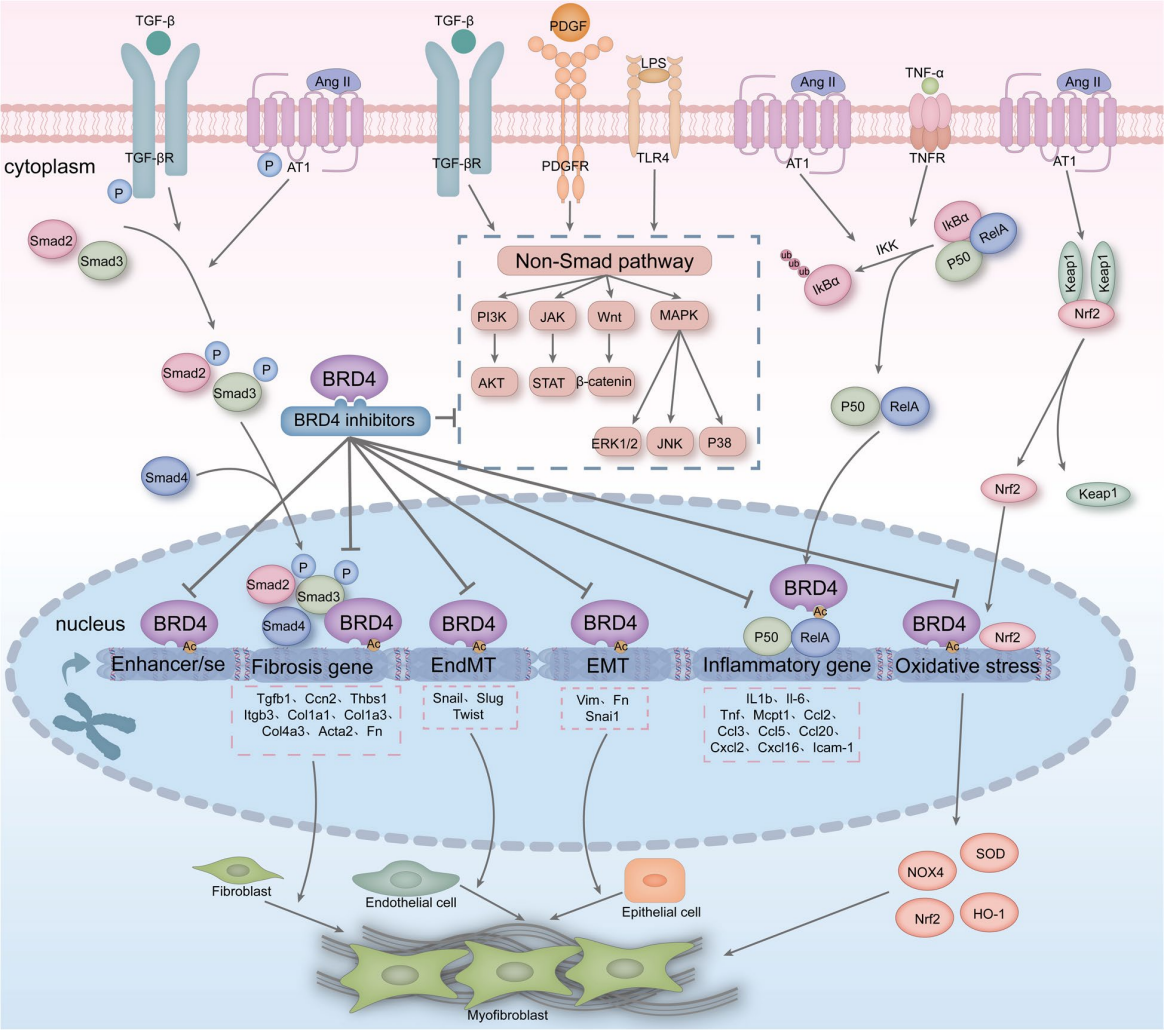
Mechanism of BRD4 involvement in organ fibrosis and mechanism of action of BRD4 inhibitors. BRD4 can bind to fibrosis-related enhancers and super-enhancers. BRD4 is involved mainly in the expression of TGF-β1-associated fibrosis genes, NF-kB-associated inflammatory genes, EMT-associated genes, EndMT-associated genes and oxidative stress-associated genes. BRD4 inhibitors primarily exert their antifibrotic effects by suppressing the expression of genes linked to the progression of fibrosis. Furthermore, BRD4 inhibitors can demonstrate anti-fibrotic properties through the suppression of the non-canonical TGF-β signaling pathway[67]

Organ fibrosis is a condition in which external stimuli lead to changes in epigenetic and gene expression within cells, changes in fibroblast morphology and function, transformation into myofibroblasts, and excessive synthesis of ECM by myofibroblasts, leading to structural damage and dysfunction of organs. Epigenetics is significantly involved in the pathogenesis of organ fibrosis [31], and BRD4 is an epigenetic reader that translates histone modification changes caused by external stimuli into gene expression changes, thus linking external stimuli to pathological changes in cells. Given the importance of BRD4 in diseases such as cancer, inflammation and fibrosis, this review introduces current research progress on BRD4 in organ fibrosis. Importantly, multiple studies have demonstrated the significant involvement of BRD4 in fibrosis development. Utilizing BRD4 inhibitors or reducing BRD4 expression has been shown to mitigate the progression of organ fibrosis [142, 183, 213, 242, 243]. BRD4 is involved mainly in the expression of genes related to fibrosis, inflammation, EMT, EndMT, and oxidative stress during the progression of organ fibrosis and is able to bind to enhancers and super-enhancers of fibrosis-related genes, which in turn promotes the progression of fibrotic diseases. In addition, BRD4 inhibitors may also significantly slow the progression of organ fibrosis by acting on non-classical TGF-β/Smad signaling pathways, such as the PI3K/AKT, JAK/STAT, Wnt/β-catenin, and MAPK pathways [Fig. 8]. Therefore, we believe that BRD4 has potential as an anti-fibrotic target. However, the specific mechanism of action of BRD4 in the development of fibrosis is currently unknown and needs to be further explored. In current preclinical studies, BET inhibitors are mostly used to study the mechanism of BRD4 in organ fibrosis, which makes it difficult to exclude the influence of other BET proteins on the fibrotic disease process, and the use of selective inhibitors of BRD4 to study the mechanism of BRD4 in organ fibrosis may become an important direction of research in the future [261]. In addition, we present the challenges of BRD4 inhibitors in fibrotic diseases. BRD4 as an epigenetic reader, we need to focus on the function of BRD4 in normal cells and tissues under physiological conditions and explore the effects of BRD4 inhibition or absence on normal tissues and organs. Soo Young Kim et al. found that cardiomyocyte BRD4 is important for maintaining myocardial function and myocardial energy homeostasis [280]. In the liver, some researchers have found that BET proteins are involved in hepatocyte proliferation and liver regeneration [281]. The ability of the BRD4 inhibitor JQ1 to significantly inhibit hepatocyte regeneration suggests that we need to pay attention to the timing of the use of JQ1 in the treatment of hepatic fibrotic diseases and to avoid the use of JQ1 in the stage of rapid hepatocyte regeneration[282]. Similarly, Julia Wilflingseder et al. and Janina Schreiber et al. found that BRD4 is involved in renal growth, development, and repair and that the use of the BET inhibitor JQ1 may lead to renal hypoplasia. The administration of JQ1 before renal injury inhibited renal repair and led to renal failure, whereas the administration of JQ1 2–7 days after renal injury inhibited excessive renal repair and ameliorated renal interstitial fibrosis, suggesting that BET inhibitors may have potential teratogenic effects [283, 284]. In summary, in clinical studies, we need to focus on the duration of dosing and the teratogenic effects of BRD4 inhibitors. On the basis of the current understanding of BRD4 in organ fibrosis, some researchers have begun to explore the combined effects of BRD4 inhibitors and other drugs. Raghda Hassan et al. found that JQ1 and atorvastatin synergistically inhibited hepatic stellate cell activation [285]. However, Hyunkyung Jung et al. showed that the BRD4 inhibitor JQ1 has an antagonistic effect on FXR agonists (OCA) [286]. These findings suggest that we need to be equally aware of the use of BRD4 inhibitors in combination with other drugs.

Approximately 1700 BRD4 inhibitors are available, and only a dozen BRD4 inhibitors have been used in organ fibrosis studies. Pelabresib, a selective inhibitor of BRD4, is in phase III clinical trials [287] for the treatment of myelofibrosis (NCT04603495), but no relevant studies have reported the therapeutic role of Pelabresib in organ fibrosis. RVX-208, a BET inhibitor, is in clinical phase III studies for the improvement of cardiovascular disease and has been reported to have an ameliorative effect on organ fibrosis in preclinical studies, and it is expected to be a potential drug for the treatment of organ fibrosis. In addition, CPI-0610 [288, 289], BMS-986158 (NCT05372354, NCT04817007) [290], BMS-986378 [291], ABBV-075 [292, 293], ABBV-744 [294, 295], and other BRD4 inhibitors are also in the clinical research stage. Fibrosis is an over-repairing disease that is difficult to reverse, and there are no clinically available drugs for treating cardiac, hepatic or renal fibrotic diseases. Various BRD4 inhibitors have shown promising antifibrotic effects in preclinical studies. Appropriate doses for the treatment of fibrotic diseases can be found on the basis of available clinical trial data on BRD4 inhibitors, which are used in subsequent fibrotic clinical trials. If BRD4 inhibitors show promising therapeutic effects in clinical studies of fibrosis, this would be a major breakthrough in the treatment of fibrotic disease and could lead to significant cost savings in fibrotic drug development. In addition, the green channel mechanism for orphan drugs for fibrotic diseases will significantly shorten the review and approval cycle for fibrotic therapeutics, and if BRD4 inhibitors have the effect of reversing or slowing the progression of fibrosis in clinical trials, they will provide more options for the treatment of fibrosis in the clinic.

## Data Availability

No datasets were generated or analysed during the current study.

## Abbreviations

AB: Aortic banding
AIH: Autoimmune hepatitis
AKI: Acute kidney injury
AKT: Protein kinase B
ALD: Alcohol-associated liver disease
ALT: Alanine transaminase
Ang II: Angiotensin II
ANP: Atrial natriuretic peptide
AST: Aspartate transaminase
BALF: Bronchoalveolar lavage fluid
BD: Bromodomains
BEL: Bellidifolin
BET: Bromodomain and extra-terminal structural domain
BID: Base-interacting structural domain
BLM: Bleomycin
BNP: Brain natriuretic peptide
BRD: Bromodomain
BRD2: Bromodomain-containing protein 2
BRD3: Bromodomain-containing protein 3
BRD4: Bromodomain-containing protein 4
BRD4-HAT: Histone acetyltransferase activity of BRD4
BRDT: Bromodomain testis-specific protein
CAFs: Cancer-associated fibroblasts
CDK9: Cycle-dependent kinase 9
CFs: Cardiac fibroblasts
CKD: Chronic kidney disease
CCL2: C-C motif chemokine ligand 2
CCL5: C-C motif chemokine ligand 5
CCL20: C-C motif chemokine ligand 20
Cd: Cadmium
COL I: Collagen I
COL III: Collagen III
COL IV: Collagen IV
COPD: Chronic obstructive pulmonary disease
CPS: C-terminal phosphorylation site
CTD: C-terminal domain
CTGF: Connective tissue growth factor
CTM: C-terminal motif
CXCL-16: Chemokine (C-X-C motif) ligand 6
CXCL6: Chemokine (C-X-C motif) ligand 6
Cyc T1: Cycle T1
DCM: Dilated cardiomyopathy
DKD: Diabetic kidney disease
ECM: Extracellular matrix
EGFR: Epidermal growth factor receptor
Egr1: Early-immediate gene
EMT: Epithelial-mesenchymal transition
EndMT: Endothelial-to-mesenchymal transition
ERK1/2: Extracellular signal regulated kinases ½
ERS: Endoplasmic reticulum stress
ET: Extra-terminal
ETS1: ETS proto-oncogene 1
EZH2: Enhancer of zeste homolog 2
FN: Fibronectin
FoxM1: Forkhead box M1
FOXO4: Forkhead box protein O4
FSGS: Focal segmental glomerulosclerosis
FX: Fucoxanthin
GN: Glomerulonephritis
HAT: Histone acetyltransferases
HBV: Hepatitis B virus
HCC: Hepatocellular carcinoma
HCV: Hepatitis C virus
HDAC: Histone deacetylases
HO-1: Heme oxygenase-1
HG: High-glucose
HMGB1: High mobility group box 1
HN: Hypertensive nephropathy
hSAECs: Human small airway epithelial cell lines
HR: Hypoxia-reoxygenation
HSCs: Hepatic stellate cells
HUVECs: Human umbilical vein endothelial cells
ICAM-1: Intercellular adhesion molecule
IFN-γ: Interferon-γ
IL-6: Interleukin-6
IL-1β: Interleukin-1β
IL-8: Interleukin-8
IL-17: Interleukin-17
iNOS: Inducible nitric oxide synthase
IPF: Idiopathic pulmonary fibrosis
IRI: Ischemia-reperfusion injury
ISO: Isoprenaline
Itgb3: Integrin β3
JNK: C-Jun N-terminal protein kinase
LFs: Lung fibroblasts
LPS: Lipopolysaccharide
MAECs: Mouse aortic endothelial cells
MAPK: Mitogen-activated protein kinase
MCP-1: Monocyte chemotactic protein-1
MI: Myocardial infarction
NASH: Non-alcoholic steatohepatitis
NOX4: NADPH oxidase 4
NLRP3: NOD-like receptor protein 3
NPS: N-terminal phosphorylation site
NRCFs: Neonatal rat cardiac fibroblasts
Nrf2: Nuclear factor erythroid 2-related factor 2
NTS: Nephrotoxic serum
PAH: Pulmonary arterial hypertension
PAL-1: Plasminogen activator inhibitor-1
PARP1: Poly (ADP-ribose) polymerase-1
PBC: Primary biliary cholangitis
PDGF: Platelet-derived growth factor
PDGFR: Platelet-derived growth factor receptor
PID: P-TEFb interaction domain
PLK1: Polo-like kinase 1
PPAR-γ: Peroxisome proliferator-activated receptor-γ
P-TEFb: Positive transcription elongation factor b
RCC: Renal cell carcinoma
rCDE: Repetitive mucosal cat dander extract
RILF: Radiation-induced lung fibrosis
RIPK-1: Receptor-interacting protein kinase 1
RNA Pol II: RNA polymerase II
ROS: Reactive oxygen species
SA: Simplicity acid
SAA: Salvianolic acid A
α-SMA: α-Smooth muscle actin
SIRT1: Silent information regulator 1
SMAD3: SMAD family member 3
SNAI1: Snail family transcriptional repressor 1
SnRNP: Small nuclear ribonucleoprotein
SOD: Superoxide dismutase
SOX9: Sex-determining region Y-box 9
SRF: Serum response factor
STAT3: Signal transducer and activator of transcription 3
TAC: Transverse aortic constriction
TFs: Transcription factors
TGF-β: Transforming growth factor β
Thbs1: Thrombospondin 1
TIMP1: Tissue inhibitor of metalloproteinase 1
TNF-α: Tumor necrosis factor-α
UUO: Unilateral ureteral obstruction
VIM: Vimentin
